# One-Pot Difunctionalization of Aryldiazonium Salts for Synthesis of *para*-Azophenols

**DOI:** 10.3389/fchem.2022.818627

**Published:** 2022-01-26

**Authors:** Zhenhua Liu, Yang Fang, Yi Liu, Wei Fu, Xingxing Gan, Wen Gao, Bo Tang

**Affiliations:** ^1^ Key Laboratory of Molecular and Nano Probes, Shandong Provincial Key Laboratory of Clean Production of Fine Chemicals, College of Chemistry, Chemical Engineering and Materials Science, Collaborative Innovation Center of Functionalized Probes for Chemical Imaging in Universities of Shandong, Ministry of Education, Shandong Normal University, Jinan, China; ^2^ Department of Pharmacy, Zibo Central Hospital, Zibo, China

**Keywords:** aryldiazonium salts, difunctionalization, *p*-azophenols, one-step, metal-free

## Abstract

A novel difunctionalization of aryldiazonium salts was realized for the one-step generation of symmetric and asymmetric *p*-azophenols. This approach is proceeded by the sequentially regioselective aromatic C-O and C-N bond construction under mild reaction conditions, unlocking a new reaction strategy to facilitate the synthesis of *p*-azophenols.

## Introduction

Azoaromatic compounds are significant core motifs and found the prevalence in a variety of applications due to their unique properties of the azo unit (Pang et al., 2020). They are universally applied in synthetic chemistry (Nguyen et al., 2018), sensing probes (Dong et al., 2015), organic dyes (Bafana et al., 2011), smart materials (Mohamed et al., 2015; Gerkman et al., 2020), and therapeutic agents (Lizard et al., 2020; Babamale et al., 2021). Considering the widespread utility of aromatic azo scaffolds, a handful of methods for their formation were established by adding an external oxidant or noble metal catalyst (Grirrane et al., 2008; Dai et al., 2018; Han et al., 2021; Sitter and Vannucci, 2021). Therefore, the synthesis route to achieve azobenzene derivatives in an efficient and handy manner continues to be keenly pursued.

**FIGURE 1:**
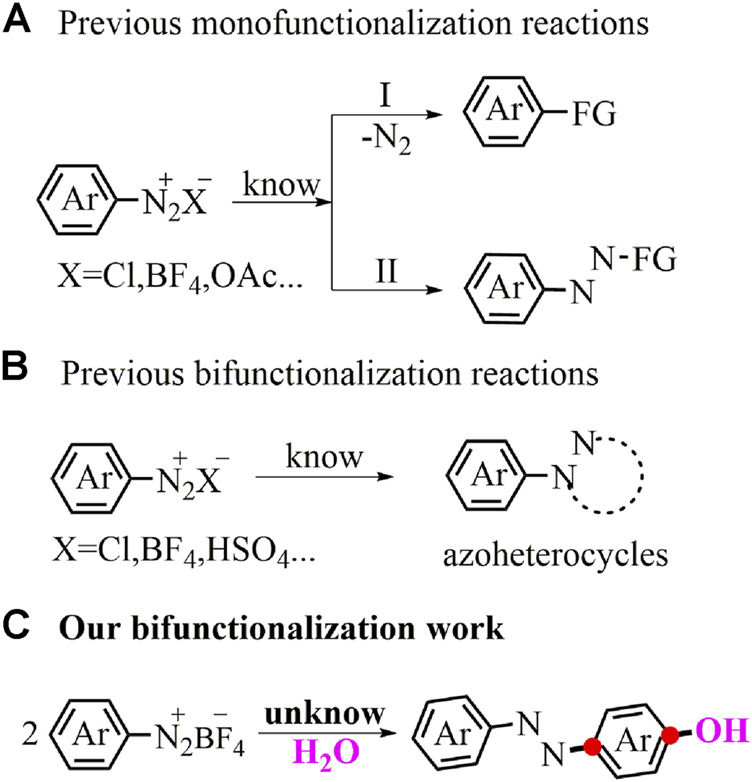
Functionalizations of aryldiazonium salts.

Aryldiazonium salts (Griess, 1858), abundant and inexpensive building blocks, have stimulated a series of remarkable name reactions as useful and efficient synthons over the next hundred years (Sandmeyer, 1884; Hodgson, 1947; Gomberg and Bachmann, 1924; Balzand Schiemann, 1927; Meerwein et al., 1939; Kikukawa and Matsuda, 1977; Kikukawa et al., 1979). Even to this day, the aryldiazonium compounds still receive much attention and constantly result in plenty of new functionalization conversions. For example, the diazo-oriented substitutions of aryldiazonium salts with various coupling partners *via* the denitrogenation step have been successfully explored (Figure 1A) (Mo et al., 2018; Felpin and Sengupta, 2019; Yuan et al., 2021). Aryldiazonium salts by the nucleophilic addition-type reaction have also been extensively used for inserting the azo group into the target molecules (Figure 1A) (Liu F et al., 2017; Liu Z et al., 2017; Abrams et al., 2018; Liu et al., 2018). Recently, cyclization by retaining the privileged N_2_ fragment has been a thriving pattern in heterocyclic chemistry (Figure 1B) (Mo et al., 2013; Zhang et al., 2020). Apart from the familiar aryl or/and aryl azo precursors described above, the aryl-based difunctionalization pattern of aryldiazonium salts has seriously remained in the shadow of underdevelopment. Herein, we exposed a first example utilizing this aryl-based difunctionalization tactics of aryldiazonium salts with H_2_O to directly assemble versatile *p*-azophenols in good yields under mild reaction conditions (Figure 1C).

## Results and Discussion

To optimize our aryl difunctionalization scenario, we first employed phenyldiazonium salt **1a** as the standard substrate to evaluate reaction conditions with the high-yielding product 4-(phenyldiazenyl)phenol **2a** in mind. When MeOH was conducted as a solvent in the difunctionalization reaction of the phenyldiazonium salt **1a**, an unprecedented preference was smoothly delivered with the yielding product **2a** in 46% yield (Table 1, entry 1). The absolute structure of **2a** was also confirmed by X-ray diffraction analysis (CCDC No. 2070994, see details in SI). In light of this initial experimental result, we further investigated other solvents for this aryl-based cascade reaction, while keeping the moderate yield permanent (Table 1, entries 2–5). With the consideration of a hydroxyl source in the target product, we next screened the amount of H_2_O in the reaction (Table 1, entries 6–8). The optimal ratio of MeOH/H_2_O was found to be 3:1 with a superior increase in the yield to 89%. Then, other bases were probed in the reaction conditions and gave somewhat unsatisfied yields of the desired product (Supplementary Table S1, entries 1-8 in SI). Similarly, adjusting the amount of the base or reaction temperature was proved to impede the desired conversion (Supplementary Table S1, entries 9–11 in SI).

**TABLE 1:** Optimization of the reaction conditions.^a^

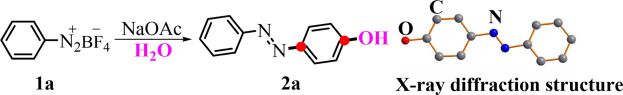

**TABLE 1:** Continued.

Entry	Base	Solvent	Yield/%^a^
1	NaOAc	MeOH	46
2	NaOAc	Ethanol	42
3	NaOAc	MeCN	40
4	NaOAc	Acetone	41
5	NaOAc	DCM	0
6	NaOAc	MeOH/H_2_O = 1:1	80
7	NaOAc	MeOH/H_2_O = 3:1	89
8	NaOAc	MeOH/H_2_O = 9:1	63

a Conditions: 1a (0.5 mmol, 1.0 eq.), Base (0.5 mmol, 1 eq.), in 2 ml solvent at 0°C-r.t. for 3 h; yields of isolated products.

**SCHEME 1:**
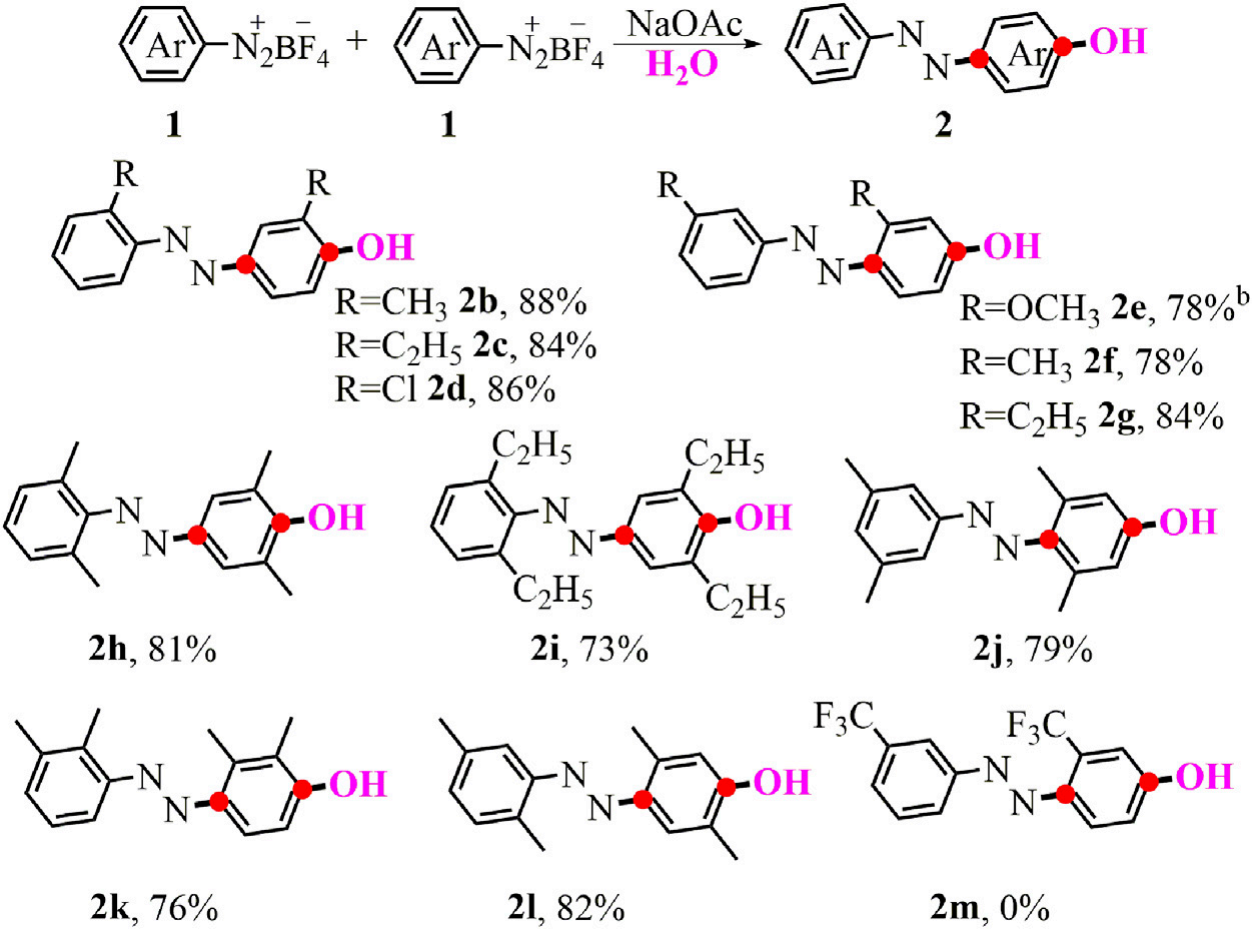
Synthesis of symmetrical *p*-azophenol. ^a^Conditions: 1 (0.5 mmol), NaOAc (0.5 mmol, 1 eq.), in 2 ml of the mixed solvent (V_MeOH_/V_H2O_ = 3:1) at 0°C-r.t. for 3 h; yields of isolated products. ^b^Without 1.0 eq. NaOAc.

Having the optimized reaction conditions in hand, we began examining the reactivity of various aryldiazonium salts to give the corresponding *p*-azophenol derivatives (Scheme 1). Aryldiazonium salts with *ortho*- and *meta*-substituents including electron-donating or electron-withdrawing groups (**1b**-**1g**) smoothly reacted with H_2_O to produce the symmetrical products (**2b**-**2g**) in good yields. In addition, poly-substituents with different positions on the aryldiazonium salts were fully compatible in the standard conditions, delivering azo-containing phenolic compounds (**2h**-**2l**) in 73–82% yields. Regrettably, when the substrate bearing the *meta*-trifluoromethyl group was performed in the standard tandem reaction, no desired difunctionalized product **2m** was observed.

**SCHEME 2:**
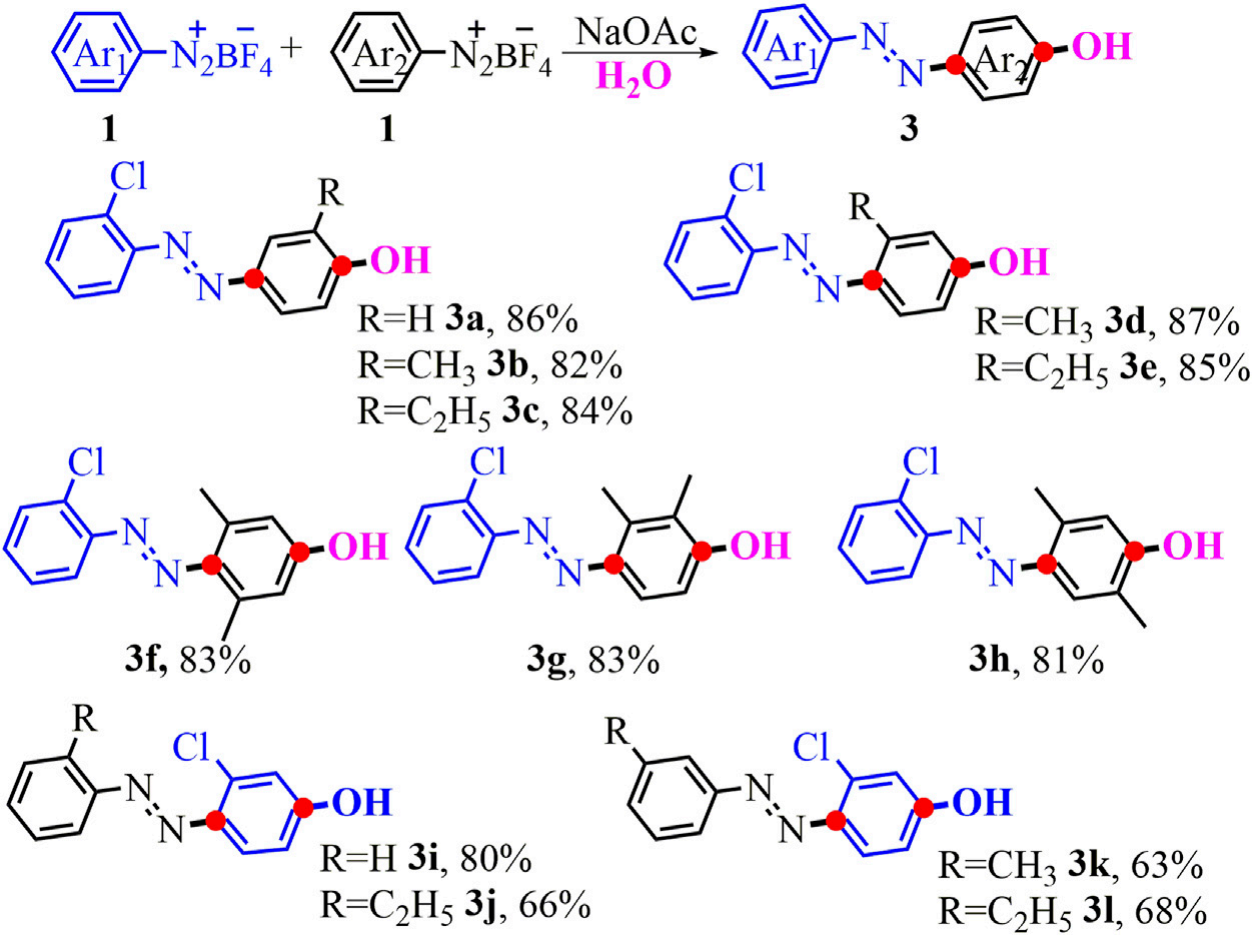
Synthesis of unsymmetrical *p*-azophenol. ^a^Conditions: 1 (0.5 mmol), NaOAc (0.5 mmol, 1 eq.), in 2 ml of the mixed solvent (V_MeOH_/V_H2O_ = 3:1) at 0°C-r.t. for 3 h; yields of isolated products.

A cross reaction between two different diazonium salts by using this efficient difunctionalization procedure is undoubtedly an attractive extension (Scheme 2). Just as we speculated, the reaction with the phenyldiazonium salt **1a** and 2-chlorobenzene diazonium salt **1d**was viable in this cascade conversion and provided the unsymmetrical *p*-azophenol **3a** in 86% yield. Then, we further examined other cross-coupling partners with the 2-chlorobenzene diazo salt as one model reactant. To our delight, aryldiazonium salts attached to the *ortho*- or *meta*-group were smoothly subjected into the reaction, yielding the aimed unsymmetrical products (**3b**-**3e**) in 82–87% yields. Similarly, the substrate with multiple substituents at different positions was also found to successfully deliver the target products (**3f-3g)**. The X-ray structure of products **3f** and **3g** (CCDC No. 2070995; 2070996, see details in SI) also validated the asymmetrical structure of the assumed product. Note that the 3-chlorobenzene diazonium salt by combining with electron-neutral or electron-donating ones was well suitable in the reaction, producing the unsymmetrical *p*-azophenol (**3i-3l**) in 63–80% yields.

**SCHEME 3:**
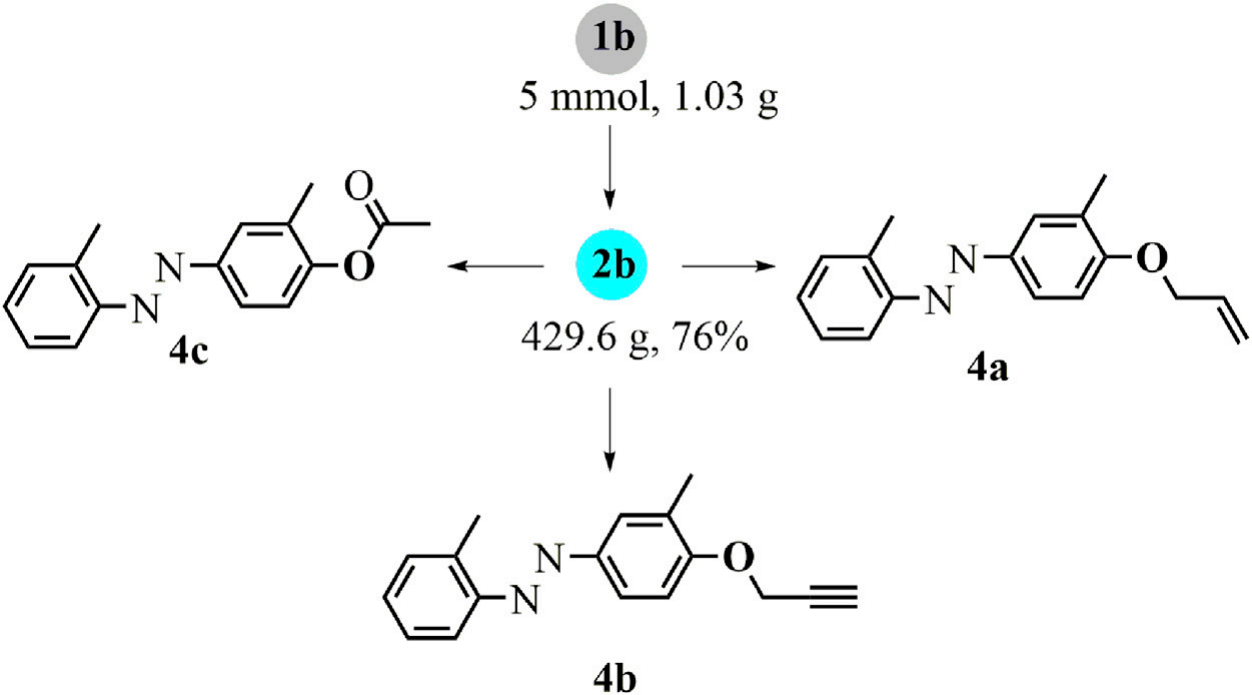
Large-scale reaction and derivatizations.

To improve the scalability and practicality of this difunctionalization approach, large-scale synthesis was applied with the substrate **1b** in the standard one-pot reaction, leading to the *p*-azophenol derivative **2b** with a satisfied yield (Scheme 3). Regarding the possibilities for further decoration of the hydroxyl group, this difunctionalized product **2b** was readily introduced into allylation (**4a**), propargylation (**4b**), and acetylation (**4c**) with the corresponding modification reagents.

To obtain the information of this difunctionalization pathway, free radical trap experiments (Liu et al., 2019; Liu et al., 2020) with using 2,2,6,6-tetramethyl-1-piperidinyloxy (TEMPO) or 2,6-di-tert-butyl-4-methylphenol (BHT) did not give the target product **2a** or the corresponding radical coupling byproduct with the assistance of HRMS (Supplementary Scheme S1A in SI). Those inhibited results might ascribe to the acidity of BHT (Huang and Lumb, 2019) or oxidability of TEMPO (Ryan et al., 2019) influencing on the stability of diazonium salts in the standard reaction. So an ionic process was still heavily weighted in the optimal process. To examine the sequence of hydroxylation and azo-modification, a hypothesized PhOH intermediate led to the *para*-functionalization product **2b**’ rather than the target product **2b** (Supplementary Scheme S1B in SI). Diphenylazo **5a** (Okumura et al., 2013) replacing the diazonium salt was used as the reactant whereas the desired formation of **2a** was completely shut down (Supplementary Scheme S1C in SI). Those observations were more likely to highlight that the diazo-localized hydroxylation of the aryldiazonium salt preceded over the *para*-selective azo reaction in the standard reaction. In order to further help understand the reaction mechanism, the charge densities of the carbon adjacent to the azo group in the aryldiazonium cation were calculated by the natural population analysis (NPA) charge analysis (see Supplementary Scheme S2 in SI).

**SCHEME 4:**
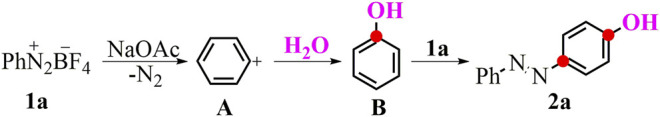
Possible mechanism for the reaction.

In view of the above experimental observations and the results of calculated NPA charges for selective carbon in aryl azo ions (see Supplementary Scheme S2 in SI), a proposed pathway is displayed in Scheme 4. Initially, the releasing nitrogen of substrate **1a** produced the active phenyl cation **A** (Canning et al., 1999; Canning et al., 2002), which quickly underwent the electrophilic substitution reaction with H_2_O by forming the phenol intermediate **B** (Ando, 1978). If different diazonium salts were employed, the substrate bearing more positive charges at the carbon adjacent to the azo group could be preferentially converted to phenol species. Sequentially, the *in situ para*-selective bias of species **B** with another diazonium salt through electrophilic aromatic substitution (EArS) occurred (Fiege et al., 2000; Xu and Zeng, 2010; Huang and Lumb, 2019), offering the final product **2/3** in one-pot.

## Conclusion

In summary, we have successfully demonstrated a novel and practical aryl-based difunctionalization strategy of aryldiazonium salts with H_2_O, providing a convenient avenue to valuable symmetric/asymmetric *p*-azophenols in a one-pot operation. Obviously, this tandem method directly forming aromatic C-O and C-N bonds results in tolerance of readily available substrates, step efficiency, and practical reaction operations. Mechanistically, an ionic mechanism was suggested by relying on the preliminary findings of our designed experiments and the corresponding calculated NPA charges. As far as we know, this is the first example by achieving the aryl-based difunctionalization strategy of aryldiazonium salts with H_2_O to straightforwardly construct versatile *p*-azophenol derivatives in one-pot. Our difunctionalization model of diazosalts undoubtedly presents a seductive blueprint to assess the significant skeleton in organic synthetic chemistry.

### Experiment

#### General Information

All reagents were purchased from commercial sources and used without treatment, unless otherwise indicated. The products were purified by column chromatography over silica gel. ^1^H NMR and ^13^C NMR spectra were recorded at 25°C on a Varian 400 and 100 MHz, respectively, and TMS was used as the internal standard. Mass spectra were recorded on a BRUKER AutoflexIII Smartbeam MS-spectrometer. High-resolution mass spectra (HRMS) were recorded on Bruck microTof by using the ESI method.

General procedure for the synthesis of 2/3: Phenyldiazonium tetrafluoroborate 1 (0.5 mmol, 1.0 eq.) and sodium acetate (0.5 mmol, 1.0 eq.) were added in an oven-dried 15-ml Schlenk tube with 2 ml of mixed solvent (MeOH/H_2_O = 3/1) at 0°C; the reaction mixture was then stirred at r.t. for 3 h. The resulting reaction mixture was taken up by dichloromethane (3 × 15 ml). The organic layer was washed with brine (3 × 40 ml), dried over MgSO_4_, and concentrated under reduced pressure. The residue was purified by a silica gel column chromatography (petroleum ether/ethyl acetate = 5:2), and the target product two or three was afforded.

(E)-4-(phenyldiazenyl)phenol (2a). Compound 2a was prepared in a manner of the general procedure for the synthesis of 2/3. Yield 89%, yellow solid. ^1^H NMR (400 MHz, ^CDCl^3) *δ* 7.80 (d, *J* = 8.6 Hz, 4H), 7.50–7.33 (m, 3H), 6.86 (d, *J* = 8.6 Hz, 2H), and 5.21 (s, 1H); ^13^C NMR (100 MHz, ^CDCl^3) *δ* 157.3, 151.6, 146.0, 129.4, 128.0, 123.9, 121.5, and 114.7; HRMS (ESI-TOF)m/z calculated for C_12_H_9_N_2_O [M-H]－: 197.0715, found: 197.0704.

(E)-2-methyl-4-(*o*-tolyldiazenyl)phenol (2b). Compound 2b was prepared in a manner of the general procedure for the synthesis of 2/3. Yield 88%, yellow solid. ^1^H NMR (400 MHz, ^CDCl^3) δ7.78–7.74 (m, 1H), 7.73–7.70 (m, 1H), 7.57 (d, *J* = 7.8 Hz, 1H), 7.35–7.29 (m, 2H), 7.28–7.22 (m, 1H), 6.87 (d, *J* = 8.4 Hz, 1H), 5.41 (s, 1H), 2.70 (s, 3H), and 2.34 (s, 3H); ^13^C NMR (100 MHz, ^CDCl^_3_) *δ* 156.5, 150.8, 147.4, 137.4, 131.1, 130.2, 126.3, 125.6, 124.4, 123.0, 115.4, 115.1, 17.5, and 15.8; HRMS (ESI-TOF) m/z calculated for C_14_H_13_N_2_O [M-H]-: 225.1028, found: 225.1018.

(E)-2-ethyl-4-((2-ethylphenyl)diazenyl)phenol (2c). Compound 2b was prepared in a manner of the general procedure for the synthesis of 2/3. Yield 84%, yellow solid. ^1^H NMR (400 MHz, ^CDCl^_3_) *δ* 7.76–7.70 (m, 1H), 7.65–7.59 (m, 1H), 7.50 (d, *J* = 7.8 Hz, 1H), 7.33–7.23 (m, 2H), 7.20–7.14 (m, 1H), 6.73 (d, *J* = 8.4 Hz, 1H), 5.43 (s, 1H), 3.14–2.99 (m, 2H), 2.70–2.56 (m, 2H), and 1.27–1.14 (m, 6H); ^13^C NMR (100 MHz, ^CDCl^_3_) *δ* 155.0, 149.2, 146.4, 142.3, 129.5, 129.4, 128.5, 125.4, 123.6, 121.0, 23.6, 21.9, 15.3, and 12.6; HRMS (ESI-TOF) m/z calculated for C_16_H_17_N_2_O [M-H]－: 253.1341, found: 253.1320.

(E)-2-chloro-4-((2-chlorophenyl)diazenyl)phenol (2d). Compound 2d was prepared in a manner of the general procedure for the synthesis of 2/3. Yield 86%, yellow solid. ^1^H NMR (400 MHz, ^CDCl^_3_) *δ* 7.93–7.87 (m, 1H), 7.76–7.69 (m, 1H), 7.61–7.56 (m, 1H), 7.49–7.4 (m, 1H), 7.30–7.22 (m, 2H), 6.77 (d, *J* = 8.6 Hz, 1H), and 4.43 (s, 1H); ^13^C NMR (100 MHz, ^CDCl^_3_) *δ* 147.7, 145.0, 144.6, 133.5, 129.8, 129.5, 126.2, 123.7, 123.4, 118.3, 116.5, and 113.8; HRMS (ESI-TOF) m/z calculated for C_12_H_7_Cl_2_N_2_O [M-H]－: 264.9935, found: 264.9907.

(E)-3-methoxy-4-((3-methoxyphenyl)diazenyl)phenol (2e). Compound 2e was prepared in a manner of the general procedure for the synthesis of 2/3. Yield 78%, yellow solid. ^1^H NMR (400 MHz, ^CDCl^_3_) *δ* 7.68–7.58 (m, 1H), 7.38 (d, *J* = 7.8 Hz, 1H), 7.33–7.25 (m, 2H), 6.90–6.83 (m, 1H), 6.32–6.12 (m, 2H), 4.07 (s, 1H), 3.89 (s, 3H), and 3.80 (s, 3H); ^13^C NMR (100 MHz, ^CDCl^_3_) *δ* 159.1, 158.1, 153.6, 150.4, 133.9, 128.5, 117.5, 115.2, 115.1, 106.4, 104.7, 96.7, 55.0, and 54.4; HRMS (ESI-TOF) m/z calculated for C_14_H_13_N_2_O_3_ [M-H]－: 257.0926, found: 257.0911.

(E)-3-methyl-4-(*m*-tolyldiazenyl)phenol (2f). Compound 2f was prepared in a manner of the general procedure for the synthesis of 2/3. Yield 78%, yellow solid. ^1^H NMR (400 MHz, ^CDCl^_3_) *δ* 7.69–7.63 (m, 3H), 7.40–7.35 (m, 1H), 7.24 (d, *J* = 7.6 Hz, 1H), 6.79–6.75 (m, 1H), 6.72–6.68 (m, 1H), 5.30 (s, 1H), 2.70 (s, 3H), and 2.45 (s, 3H); ^13^C NMR (100 MHz, ^CDCl^_3_) *δ* 158.1, 153.1, 145.1, 141.0, 138.8, 130.9, 128.8, 123.0, 120.0, 117.3, 117.0, 113.5, 21.4, and 17.6; HRMS (ESI-TOF) m/z calculated for C_14_H_13_N_2_O [M-H]－: 225.1028, found: 225.1003.

(E)-3-ethyl-4-((3-ethylphenyl)diazenyl)phenol (2g). Compound 2g was prepared in a manner of the general procedure for the synthesis of 2/3. Yield 84%, yellow solid. ^1^H NMR (400 MHz, ^CDCl^_3_) *δ* 7.66–7.57 (m, 2H), 7.53–7.46 (m, 1H), 7.34–7.21 (m, 1H), 7.19–7.08 (m, 1H), 6.70–6.62 (m, 1H), 6.59–6.50 (m, 1H), 4.98 (s, 1H), 3.12–2.86 (m, 2H), 2.63 (q, *J* = 7.6 Hz, 2H), and 1.23–1.10 (m, 6H); ^13^C NMR (100 MHz, ^CDCl^_3_) *δ* 157.6, 152.0, 146.0, 144.2, 143.3, 128.8, 127.9, 121.4, 118.6, 116.1, 114.8, 112.7, 27.7, 23.6, 15.0, and 14.4; HRMS (ESI-TOF) m/z calculated for C_16_H_17_N_2_O [M-H]－: 253.1341, found: 253.1311.

(E)-4-((2,6-dimethylphenyl)diazenyl)-2,6-dimethylphenol (2h). Compound 2h was prepared in a manner of the general procedure for the synthesis of 2/3. Yield 81%, yellow solid. ^1^H NMR (400 MHz, ^CDCl^_3_) *δ* 7.52 (s, 2H), 7.01 (s, 3H), 5.00 (s, 1H), 2.25 (s, 6H), and 2.21 (s, 6H); ^13^C NMR (100 MHz, ^CDCl^_3_) *δ* 154.1, 150.9, 145.4, 128.9, 127.8, 126.3, 122.5, 122.2, 17.4, and 14.9; HRMS (ESI-TOF) m/z calculated for C_16_H_17_N_2_O [M-H]－: 253.1341, found: 253.1306.

(E)-4-((2,6-diethylphenyl)diazenyl)-2,6-diethylphenol (2i). Compound 2i was prepared in a manner of the general procedure for the synthesis of 2/3. Yield 73%, yellow solid. ^1^H NMR (400 MHz, ^CDCl^_3_) *δ* 7.56 (s, 2H), 7.13–7.04 (m, 3H), 5.00 (s, 1H), 2.68–2.61 (m, 4H), 2.59–2.51 (m, 4H), 1.28–1.23 (m, 6H), and 1.10–1.03 (m, 6H); ^13^C NMR (100 MHz, ^CDCl^_3_) *δ* 153.2, 150.7, 145.8, 134.6, 128.6, 126.3, 126.1, 120.4, 23.8, 22.0, 14.3, and 12.6; HRMS (ESI-TOF) m/z calculated for C_20_H_25_N_2_O [M-H]－: 309.1967, found: 309.1942.

(E)-4-((3,5-dimethylphenyl)diazenyl)-3,5-dimethylphenol (2j). Compound 2j was prepared in a manner of the general procedure for the synthesis of 2/3. Yield 79%, yellow solid. ^1^H NMR (400 MHz, ^CDCl^_3_) *δ* 7.39 (s, 2H), 7.02 (s, 1H), 6.49 (s, 2H), 5.21 (s, 1H), and 2.38–2.30 (m, 12H); ^13^C NMR (100 MHz, ^CDCl^_3_) *δ* 155.5, 153.2, 144.9, 138.7, 134.4, 132.0, 120.0, 115.8, 21.3, and 19.7; HRMS (ESI-TOF) m/z calculated for C_16_H_17_N_2_O [M-H]－: 253.1341, found: 253.1333.

(E)-4-((2,3-dimethylphenyl)diazenyl)-2,3-dimethylphenol (2k). Compound 2k was prepared in a manner of the general procedure for the synthesis of 2/3. Yield 76%, yellow solid. ^1^H NMR (400 MHz, ^CDCl^_3_) *δ* 7.48–7.42 (m, 1H), 7.40–7.31 (m, 1H), 7.14 (d, *J* = 7.2 Hz, 1H), 7.10–7.03 (m, 1H), 6.63–6.55 (m, 1H), 5.04 (s, 1H), 2.62 (s, 3H), 2.55 (s, 3H), 2.29 (s, 3H), and 2.18 (s, 3H); ^13^C NMR (100 MHz, ^CDCl^_3_) *δ* 154.8, 150.3, 144.8, 138.6, 137.0, 135.2, 130.2, 124.6, 122.0, 113.4, 112.5, 111.9, 18.9, 12.7, 12.2, and 10.6; HRMS (ESI-TOF) m/z calculated for C_16_H_17_N_2_O [M-H]－: 253.1341, found: 253.1336.

(E)-4-((2,5-dimethylphenyl)diazenyl)-2,5-dimethylphenol (2l). Compound 2l was prepared in a manner of the general procedure for the synthesis of 2/3. Yield 82%, yellow solid. ^1^H NMR (400 MHz, ^CDCl^_3_) *δ* 7.45 (s, 1H), 7.31 (s, 1H), 7.15–7.02 (m, 2H), 6.63 (s, 1H), 5.03 (s, 1H), 2.60 (d, *J* = 3.0 Hz, 6H), 2.29 (s, 3H), and 2.19 (s, 3H); ^13^C NMR (100 MHz, ^CDCl^_3_) *δ* 155.2, 149.9, 144.3, 137.2, 134.8, 133.3, 129.9, 129.7, 120.9, 117.5, 115.6, 115.1, 20.0, 16.2, and 14.5; HRMS (ESI-TOF) m/z calculated for C_16_H_17_N_2_O [M-H]－: 253.1341, found: 253.1313.

(E)-4-((2-chlorophenyl)diazenyl)phenol (3a). Compound 3a was prepared in a manner of the general procedure for the synthesis of 2/3. Yield 86%, yellow solid. ^1^H NMR (400 MHz, ^CDCl^_3_) *δ* 7.94–7.80 (m, 2H), 7.62–7.54 (m, 1H), 7.51–7.43 (m, 1H), 7.34–7.20 (m, 2H), 6.97–6.78 (m, 2H), and 5.44 (s, 1H); ^13^C NMR (100 MHz, ^CDCl^_3_) *δ* 157.7, 147.7, 146.3, 133.6, 130.0, 129.5, 126.2, 124.5, 116.5, and 114.8; HRMS (ESI-TOF) m/z calculated for C_12_H_8_ClN_2_O [M-H]－: 231.0325, found: 231.0315.

(E)-4-((2-chlorophenyl)diazenyl)-2-methylphenol (3b). Compound 3b was prepared in a manner of the general procedure for the synthesis of 2/3. Yield 82%, yellow solid. ^1^H NMR (400 MHz, ^CDCl^_3_) *δ* 7.76–7.66 (m, 2H), 7.63–7.53 (m, 1H), 7.49–7.42 (m, 1H), 7.30–7.21 (m, 2H), 6.80 (d, *J* = 8.4 Hz, 1H), 5.49 (s, 1H), and 2.26 (s, 3H); ^13^C NMR (100 MHz, ^CDCl^_3_) *δ* 156.2, 147.8, 146.2, 133.5, 129.8, 129.5, 126.2, 124.8, 123.6, 122.8, 116.5, 114.2, and 14.8; HRMS (ESI-TOF) m/z calculated for C_13_H_10_ClN_2_O [M-H]－: 245.0482, found: 245.0445.

(E)-4-((2-chlorophenyl)diazenyl)-2-ethylphenol (3c). Compound 3c was prepared in a manner of the general procedure for the synthesis of 2/3. Yield 84%, yellow solid. ^1^H NMR (400 MHz, ^CDCl^_3_) *δ* 7.75 (s, 1H), 7.68 (d, *J* = 8.4 Hz, 1H), 7.62–7.51 (m, 1H), 7.50–7.40 (m, 1H), 7.32–7.18 (m, 2H), 6.79–6.53 (m, 1H), 5.46 (s, 1H), 2.63 (q, *J* = 7.4 Hz, 2H), and 1.27–1.10 (m, 3H); ^13^C NMR (100 MHz, ^CDCl^_3_) *δ* 155.8, 147.8, 146.3, 133.4, 129.8, 129.7, 129.5, 126.2, 123.8, 122.0, 116.6, 114.5, 21.9, and 12.6; HRMS (ESI-TOF) m/z calculated for C_14_H_12_ClN_2_O [M-H]－: 259.0638, found: 259.0630.

(E)-4-((2-chlorophenyl)diazenyl)-3-methylphenol (3d). Compound 3d was prepared in a manner of the general procedure for the synthesis of 2/3. Yield 87%, yellow solid. ^1^H NMR (400 MHz, ^CDCl^_3_) *δ* 7.71–7.63 (m, 1H), 7.61–7.51 (m, 1H), 7.49–7.41 (m, 1H), 7.32–7.21 (m, 2H), 6.79–6.57 (m, 2H), 5.22 (s, 1H), and 2.62 (s, 3H); ^13^C NMR (100 MHz, ^CDCl^_3_) *δ* 157.7, 148.0, 144.4, 140.6, 133.6, 129.7, 129.5, 126.1, 117.2, 116.8, 116.2, 112.8, and 16.7; HRMS (ESI-TOF) m/z calculated for C_13_H_10_ClN_2_O [M-H]－: 245.0482, found: 245.0471.

(E)-4-((2-chlorophenyl)diazenyl)-3-ethylphenol (3e). Compound 3e was prepared in a manner of the general procedure for the synthesis of 2/3. Yield 85%, yellow solid. ^1^H NMR (400 MHz, ^CDCl^_3_) *δ* 7.75–7.62 (m, 1H), 7.60–7.52 (m, 1H), 7.49–7.39 (m, 1H), 7.36–7.17 (m, 2H), 6.79–6.53 (m, 2H), 5.19 (s, 1H), 3.05 (q, *J* = 7.4 Hz, 2H), and 1.27–1.10 (m, 3H); ^13^C NMR (100 MHz, ^CDCl^_3_) *δ* 157.9, 148.0, 146.6, 143.7, 133.6, 129.7, 129.5, 126.1, 117.0, 116.7, 114.7, 112.8, 23.6, and 15.1; HRMS (ESI-TOF) m/z calculated for C_14_H_12_ClN_2_O [M-H]－: 259.0638, found: 259.0621.

(E)-4-((2-chlorophenyl)diazenyl)-3,5-dimethylphenol (3f). Compound 3f was prepared in a manner of the general procedure for the synthesis of 2/3. Yield 83%, yellow solid. ^1^H NMR (400 MHz, ^CDCl^_3_) *δ* 7.60–7.54 (m, 1H), 7.52–7.45 (m, 1H), 7.31–7.22 (m, 2H), 6.55 (s, 2H), 5.27 (s, 1H), and 2.49 (s, 6H); ^13^C NMR (100 MHz, ^CDCl^_3_) *δ* 155.5, 148.4, 143.1, 135.7, 133.9, 129.7, 129.4, 126.1, 115.9, 115.0, and 19.8; HRMS (ESI-TOF) m/z calculated for C_14_H_12_ClN_2_O [M-H]－: 259.0638, found: 259.0616.

(E)-4-((2-chlorophenyl)diazenyl)-2,3-dimethylphenol (3g). Compound 3g was prepared in a manner of the general procedure for the synthesis of 2/3. Yield 83%, yellow solid. ^1^H NMR (400 MHz, ^CDCl^_3_) *δ* 7.62–7.51 (m, 2H), 7.49–7.40 (m, 1H), 7.31–7.20 (m, 2H), 6.60 (d, *J* = 8.8 Hz, 1H), 5.26 (s, 1H), 2.61 (s, 3H), and 2.17 (s, 3H); ^13^C NMR (100 MHz, ^CDCl^_3_) *δ* 155.7, 148.2, 144.6, 139.5, 133.5, 129.59, 129.50, 126.1, 122.2, 116.9, 113.9, 112.2, 12.7, and 10.6; HRMS (ESI-TOF) m/z calculated for C_14_H_12_ClN_2_O [M-H]－: 259.0638, found: 259.0608.

(E)-4-((2-chlorophenyl)diazenyl)-2,5-dimethylphenol (3h). Compound 3h was prepared in a manner of the general procedure for the synthesis of 2/3. Yield 81%, yellow solid. ^1^H NMR (400 MHz, ^CDCl^_3_) *δ* 7.61–7.51 (m, 2H), 7.48–7.42 (m, 1H), 7.29–7.21 (m, 2H), 6.65 (s, 1H), 5.22 (s, 1H), 2.59 (s, 3H), and 2.19 (s, 3H); ^13^C NMR (100 MHz, ^CDCl^_3_) *δ* 156.3, 148.2, 144.1, 138.2, 133.4, 129.57, 129.50, 126.1, 121.3, 117.9, 116.8, 115.6, 16.2, and 14.4; HRMS (ESI-TOF) m/z calculated for C_14_H_12_ClN_2_O [M-H]－: 259.0638, found: 259.0619.

(E)-3-chloro-4-(phenyldiazenyl)phenol (3i). Compound 3i was prepared in a manner of the general procedure for the synthesis of 2/3. Yield 80%, yellow solid. ^1^H NMR (400 MHz, ^CDCl^_3_) *δ* 7.86–7.74 (m, 3H), 7.72–7.65 (m, 1H), 7.40–7.27 (m, 2H), 6.86 (d, *J* = 8.8 Hz, 2H), and 4.43 (s, 1H); ^13^C NMR (100 MHz, ^CDCl^_3_) *δ* 157.7, 152.5, 145.8, 134.0, 129.1, 129.0, 124.2, 121.0, 120.5, and 114.9; HRMS (ESI-TOF) m/z calculated for C_12_H_8_ClN_2_O [M-H]－: 231.0325, found: 231.0320.

(E)-3-chloro-4-((2-ethylphenyl)diazenyl)phenol (3j). Compound 3j was prepared in a manner of the general procedure for the synthesis of 2/3. Yield 66%, yellow solid. ^1^H NMR (400 MHz, ^CDCl^_3_) *δ* 7.83–7.62 (m, 4H), 7.42–7.29 (m, 2H), 6.84–6.74 (m, 1H), 5.28 (s, 1H), 2.71–2.57 (m, 2H), and 1.26–1.21 (m, 3H); ^13^C NMR (100 MHz, ^CDCl^_3_) *δ* 155.6, 152.6, 145.9, 134.0, 129.6, 129.0, 128.9, 123.1, 122.0, 121.0, 120.4, 114.5, 21.8, and 12.6; HRMS (ESI-TOF) m/z calculated for C_14_H_12_ClN_2_O [M-H]－: 259.0638, found: 259.0628.

(E)-3-chloro-4-(*m*-tolyldiazenyl)phenol (3k). Compound 3k was prepared in a manner of the general procedure for the synthesis of 2/3. Yield 63%, yellow solid. ^1^H NMR (400 MHz, ^CDCl^_3_) *δ* 7.85–7.74 (m, 2H), 7.67 (d, *J* = 8.8 Hz, 1H), 7.48–7.35 (m, 2H), 6.83–6.67 (m, 2H), 3.63 (s, 1H), and 2.69 (s, 3H); ^13^C NMR (100 MHz, ^CDCl^_3_) *δ* 158.7, 153.9, 144.9, 141.8, 135.0, 130.0, 129.8, 122.2, 121.5, 117.4, 117.2, 113.8, and 17.6; HRMS (ESI-TOF) m/z calculated for C_13_H_10_ClN_2_O [M-H]－: 245.0482, found: 245.0473.

(E)-3-chloro-4-((3-ethylphenyl)diazenyl)phenol (3l). Compound 3l was prepared in a manner of the general procedure for the synthesis of 2/3. Yield 68%, yellow solid. ^1^H NMR (400 MHz, ^CDCl^_3_) *δ* 7.78–7.66 (m, 2H), 7.60 (d, *J* = 8.8 Hz, 1H), 7.39–7.26 (m, 2H), 6.76–6.70 (m, 1H), 6.67–6.59 (m, 1H), 3.79 (s, 1H), 3.11–2.98 (m, 2H), and 1.25–1.18 (m, 3H); ^13^C NMR (100 MHz, ^CDCl^_3_) *δ* 158.0, 152.9, 146.7, 143.2, 133.9, 129.0, 128.8, 121.0, 120.6, 116.3, 114.7, 112.8, 23.6, and 15.1; HRMS (ESI-TOF) m/z calculated for C_14_H_12_ClN_2_O [M-H]－: 259.0638, found: 259.0627.

The dry sealed tube was charged with 2b (113.1 mg, 0.5 mmol, 1.0 eq.), allyl bromide (52.0 μL, 0.6 mmol, 1.2 eq.), and K_2_CO_3_ (138.0 mg, 1.0 mmol, 2.0 eq.) in anhydrous acetone (4 ml). Then, the reaction was heated at 50°C for 5 h. The mixture was extracted with DCM (3 × 30 ml), and the combined organic layers were dried over Na_2_SO_4_ and concentrated under reduced pressure. The residue was purified by preparative TLC (PE/EA = 5/1, v/v) to give 4a.

(E) -1-(4-(allyloxy)-3-methylphenyl)-2-(*o*-tolyl)diazene (4a). Compound 4a was prepared in a manner of the above procedure. Yield 93%, yellow solid. ^1^H NMR (400 MHz, ^CDCl^_3_) *δ* 7.84–7.72 (m, 2H), 7.64–7.53 (m, 1H), 7.34–7.19 (m, 3H), 6.91–6.83 (m, 1H), 6.15–5.95 (m, 1H), 5.51–5.38 (m, 1H), 5.34–5.23 (m, 1H), 4.67–4.53 (m, 2H), 2.69 (s, 3H), and 2.32 (s, 3H); ^13^C NMR (100 MHz, ^CDCl^_3_) *δ* 159.2, 151.0, 147.1, 137.4, 133.1, 131.1, 130.1, 127.7, 126.4, 124.2, 123.6, 117.3, 115.5, 110.9, 68.9, 17.6, and 16.5; HRMS (ESI-TOF) m/z calculated for C_17_H_18_N_2_ONa [M + Na]^+^: 289.1317, found: 289.1297.

The dry sealed tube was charged with 2b (113.1 mg, 0.5 mmol, 1.0 eq.), 3-bromopropyne (51.7 μL, 0.6 mmol, 1.2 eq.), and K_2_CO_3_ (138.0 mg, 1.0 mmol, 2.0 eq.), in anhydrous acetone (4 ml). Then, the reaction was heated at 50°C for 4.5 h. The mixture was extracted with DCM (3 × 30 ml), and the combined organic layers were dried over Na_2_SO_4_ and concentrated under reduced pressure. The residue was purified by preparative TLC (PE/EA = 5/1, v/v) to give 4b.

(F) -1-(3-methyl-4-(prop-2-yn-1-yloxy)phenyl)-2-(*o*-tolyl)diazene (4b). Compound 4b was prepared in a manner of the above procedure. Yield 92%, yellow solid. ^1^H NMR (400 MHz, ^CDCl^_3_) *δ* 7.77–7.65 (m, 2H), 7.50 (d, *J* = 7.8 Hz, 1H), 7.25–7.21 (m, 2H), 7.19–7.12 (m, 1H), 6.94 (d, *J* = 8.6 Hz, 1H), 4.68 (d, *J* = 2.4 Hz, 2H), 2.61 (s, 3H), 2.44 (t, *J* = 2.4 Hz, 1H), and 2.23 (s, 3H); ^13^C NMR (100 MHz, ^CDCl^_3_) *δ* 156.9, 149.8, 146.4, 136.4, 130.0, 129.2, 126.9, 125.3, 123.4, 122.2, 114.4, 110.3, 77.3, 74.6, 55.0, 16.4, and 15.3; HRMS (ESI-TOF) m/z calculated for C_17_H_16_N_2_ONa [M + Na]^+^: 287.1160, found: 287.1132.

The dry sealed tube was charged with 2b (113.1 mg, 0.5 mmol, 1.0 eq.), acetyl chloride (71.2 μL, 1.0 mmol, 2.0 eq.), and triethylamine (Et_3_N) (139.0 μL, 1.0 mmol, 2.0 eq.) in anhydrous DCM (4 ml). Then, the reaction was stirred at r.t. for 3 h. The mixture was extracted with DCM (3 × 30 ml), and the combined organic layers were dried over Na_2_SO_4_ and concentrated under reduced pressure. The residue was purified by preparative TLC (PE/EA = 5/1, v/v) to give 4c.

(E) -2-methyl-4-(*o*-tolyldiazenyl)phenyl acetate (4c). Compound 4c was prepared in a manner of the above procedure. Yield 95%, yellow solid. ^1^H NMR (400 MHz, ^CDCl^_3_) *δ* 7.74–7.66 (m, 2H), 7.53–7.47 (m, 1H), 7.28–7.20 (m, 2H), 7.18–7.11 (m, 1H), 7.06 (d, *J* = 8.4 Hz, 1H), 2.61 (s, 3H), 2.24 (s, 3H), and 2.18 (s, 3H); ^13^C NMR (100 MHz, ^CDCl^_3_) *δ* 167.8, 150.1, 149.7, 149.6, 136.9, 130.1, 129.9, 129.8, 125.3, 124.5, 121.5, 120.5, 114.3, 19.7, 16.4, and 15.3; HRMS (ESI-TOF) m/z calculated for C_16_H_16_N_2_O_2_Na [M + Na]^+^: 291.1109, found: 291.1084.

## Data Availability

The original contributions presented in the study are included in the article/Supplementary Material; further inquiries can be directed to the corresponding authors.
