# Supplementary material for: One-Pot Difunctionalization of Aryldiazonium Salts for Synthesis of para-Azophenols
Source: Front Chem. 2022 Jan 26;10:818627. doi: 10.3389/fchem.2022.818627 (PMC8826725; doi:10.3389/fchem.2022.818627)

***Supporting Information***

**One-Pot Difunctionalization of Aryldiazonium Salts for Synthesis of *para*-Azophenols**

Zhenhua Liu†^1^, Yang Fang†^1^, Yi Liu^1^, Wei Fu^2^, Xingxing Gan^1^, Wen Gao*^1^ and Bo Tang*^1^

^1^College of Chemistry, Chemical Engineering and Materials Science, Collaborative Innovation Center of Functionalized Probes for Chemical Imaging in Universities of Shandong, Key Laboratory of Molecular and Nano Probes, Ministry of Education, Shandong Provincial Key Laboratory of Clean Production of Fine Chemicals, Shandong Normal University, Jinan, 250014, P.R. China.

^2^Department of Pharmacy, ZiBo Central Hospital, Zibo 255000, P. R. China.

Corresponding authors

Wen Gao [gaowen@sdnu.edu.cn](mailto:gaowen@sdnu.edu.cn;%20tangb@sdnu.edu.cn)[;](mailto:gaowen@sdnu.edu.cn;%20tangb@sdnu.edu.cn)

[Bo Tang tangb@sdnu.edu.cn](mailto:gaowen@sdnu.edu.cn;%20tangb@sdnu.edu.cn)

**Contents**

**[Ⅰ. General information S2](#_Toc15528)**

**II.Optimization of the reaction conditions S2**

**[III. Mechanistic investigations S2](#_Toc15528)-S3**

**I[V. Crystallography of compounds 2a, 3f and 3g S3](#_Toc10976)-S6**

**[V. NMR spectra copies S6](#_Toc5756)-S33**

# Ⅰ. General information

All reagents were purchased from commercial sources and used without treatment, unless otherwise indicated. The products were purified by column chromatography over silica gel. ^1^H NMR and ^13^C NMR spectra were recorded at 25℃on a Varian 400 MHz and 100 MHz, respectively, and TMS was used as internal standard. Mass spectra were recorded on BRUKER AutoflexIII Smartbeam MS-spectrometer. High resolution mass spectra (HRMS) were recorded on Bruck microTof by using ESI method.

**II**.**Optimization of the reaction conditions**

Table S1 Optimization of the reaction conditions

| Entry | Base | Solvent | Yield/%^a^ |
| --- | --- | --- | --- |
| 1 | LiOAc | MeOH/H_2_O=3:1 | 78 |
| 2 | KOAc | MeOH/H_2_O=3:1 | 80 |
| 3 | CsOAc | MeOH/H_2_O=3:1 | 76 |
| 4 | Na_2_CO_3_ | MeOH/H_2_O=3:1 | 49 |
| 5 | Et_3_N | MeOH/H_2_O=3:1 | 52 |
| 6 | DIPEA | MeOH/H_2_O=3:1 | 68 |
| 7 | DBU | MeOH/H_2_O=3:1 | 51 |
| 8 | *t*-BuOK | MeOH/H_2_O=3:1 | 63 |
| 9 | NaOAc | MeOH /H_2_O=3:1 | 51^b^ |
| 10 | NaOAc | MeOH /H_2_O=3:1 | 76^c^ |
| 11 | NaOAc | MeOH/H_2_O=3:1 | 17^d^ |

^a^Conditions: 1a (0.5 mmol, 1.0 eq.), Base (0.5 mmol, 1 eq.), in 2 mL solvent at 0 °C-r.t. for 3 h; yields of isolated products. ^b^0.2 eq. NaOAc was used. ^c^5 eq. NaOAc was used. ^d^at r.t. ^e^at 0 °C. DIPEA= N,N-Diisopropylethylamine.

**III**. **Mechanistic investigations**

Scheme S1 Control experiments.

Scheme S2 Natural Population Analysis (NPA) charge analysis

The charge densities of the carbon adjacent to azo group in aryldiazonium cation is revealed as following bythe Natural Population Analysis (NPA) charge analysis at the B3LYP/6-31G**.

#

# IV. Crystallography of compounds 2a, 3f and 3g

Single-crystal X-ray diffraction data for the reported complex was recorded at a temperature of 293(2) K on a Oxford Diffraction Gemini R Ultra diffractometer, using a ω scan technique with Mo-Kα radiation (λ = 0.71073 Å). The structure was solved by Direct Method of SHELXS-97 and refined by full-matrix least-squares techniques using the SHELXL-97 program.1 Non-hydrogen atoms were refined with anisotropic temperature parameters, and hydrogen atoms of the ligands were refined as rigid groups. Basic information pertaining to crystal parameters and structure refinement is summarized in Table 1, Table 2 and Table 3.(a) G. M. Sheldrick, SHELXS-97, Program for Solution of Crystal Structures, University of Gottingen, Germany, 1997; (b) G. M. Sheldrick, SHELXL-97, Program for Refinement of Crystal Structures, University of Gottingen, Germany, 1997.

Table 1. Crystal data and structure refinement of 2a.


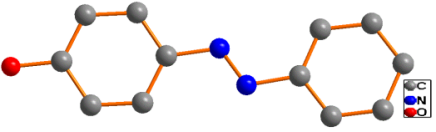


X-ray structure

CCDC: 2070994

| Empirical formula | C_12_H_10_N_2_O |
| --- | --- |
| Temperature | 293(2)K |
| Wavelength | 1.54178 Å |
| Unit cell dimensions | a = 5.8167(9) Å alpha = 90.00°  b = 10.2798(9)Å beta = 96.658(7) °  c = 8.7100(8) Ågamma = 90.00 ° |
|  | b = 16.206(3) Åbeta = 97.432(15)° |
|  | c = 10.9497(16) Ågamma = 90.00° |
| Volume | 1023.5(3) Å^3^ |
| Z | 4 |
| Calculated density | 1.286 mg/mm^3^ |
| Absorption coefficient | 0.678 mm^-1^ |
| F(000) | 416.0 |
| Crystal size | 0.18 × 0.13 × 0.03 mm^3^ |
| Theta range for data collection | 9.8 to 141.34° |
| Reflections collected / unique | 1901[R(int) = 0.0220] |
| Data / restraints / parameters | 1901 / 0 / 137 |
| Goodness-of-fit on F^2^ | 1.057 |
| Final R indices [I>2sigma(I)] | R_1_ = 0.0517, wR_2_ = 0.1332 |
| R indices (all data) | R_1_ = 0.0809, wR_2_ = 0.1648 |

Table 2. Crystal data and structure refinement of **3f**.


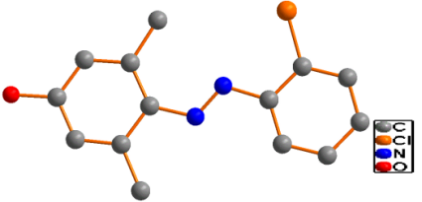


X-ray structure

CCDC: 2070995

| Empirical formula | C_14_H_13_ClN_2_O |
| --- | --- |
| Temperature | 293(2)K |
| Wavelength | 1.54178 Å |
| Unit cell dimensions | a = 21.3904(15) Å alpha = 90.00°  b = 10.2798(9)Å beta = 96.658(7) °  c = 8.7100(8) Ågamma = 90.00 ° |
|  | b = 4.0439(4) Åbeta = 91.835(7)° |
|  | c = 14.5213(11) Ågamma = 90.00° |
| Volume | 1255.46(18) Å^3^ |
| Z | 4 |
| Calculated density | 1.379 mg/mm^3^ |
| Absorption coefficient | 2.599 mm^-1^ |
| F(000) | 544.0 |
| Crystal size | 0.15 × 0.03 × 0.02mm^3^ |
| Theta range for data collection | 8.28 to 134.12° |
| Reflections collected / unique | 1901[R(int) = 0.0259] |
| Data / restraints / parameters | 2213 / 0 / 167 |
| Goodness-of-fit on F^2^ | 1.364 |
| Final R indices [I>2sigma(I)] | R_1_ = 0.0870, wR_2_ = 0.2854 |
| R indices (all data) | R_1_ = 0.1201, wR_2_ = 0.3599 |

Table 3. Crystal data and structure refinement of **3g**.


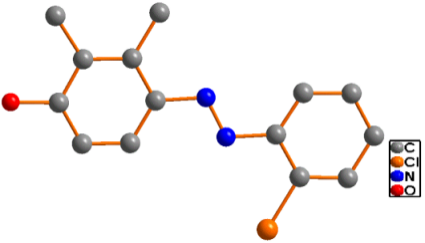


X-ray structure

CCDC: 2070996

| Empirical formula | C_14_H_13_ClN_2_O |
| --- | --- |
| Temperature | 293(2)K |
| Wavelength | 1.54178 Å |
| Unit cell dimensions | a = 13.7286(12) Å alpha = 90.00°  b = 10.2798(9)Å beta = 96.658(7) °  c = 8.7100(8) Ågamma = 90.00 ° |
|  | b = 4.0027(5) Åbeta = 94.998(8)° |
|  | c = 23.147(2) Ågamma = 90.00° |
| Volume | 1267.1(2) Å^3^ |
| Z | 4 |
| Calculated density | 1.367 mg/mm^3^ |
| Absorption coefficient | 2.575 mm^-1^ |
| F(000) | 544.0 |
| Crystal size | 0.28 × 0.04 × 0.02mm^3^ |
| Theta range for data collection | 7.22 to 134.1 ° |
| Reflections collected / unique | 1901[R(int) = 0.0535] |
| Data / restraints / parameters | 3241 / 1 / 332 |
| Goodness-of-fit on F^2^ | 1.143 |
| Final R indices [I>2sigma(I)] | R_1_ = 0.0678, wR_2_ = 0.1999 |
| R indices (all data) | R_1_ = 0.0991, wR_2_ = 0.2182 |

# V. NMR spectra copies


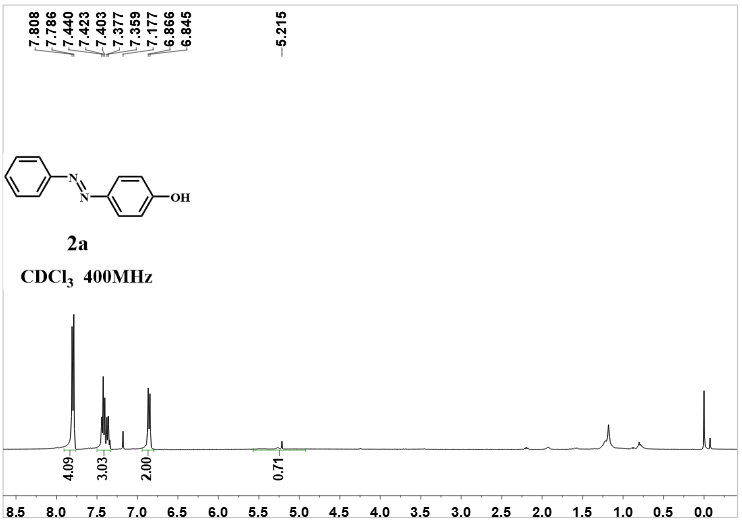


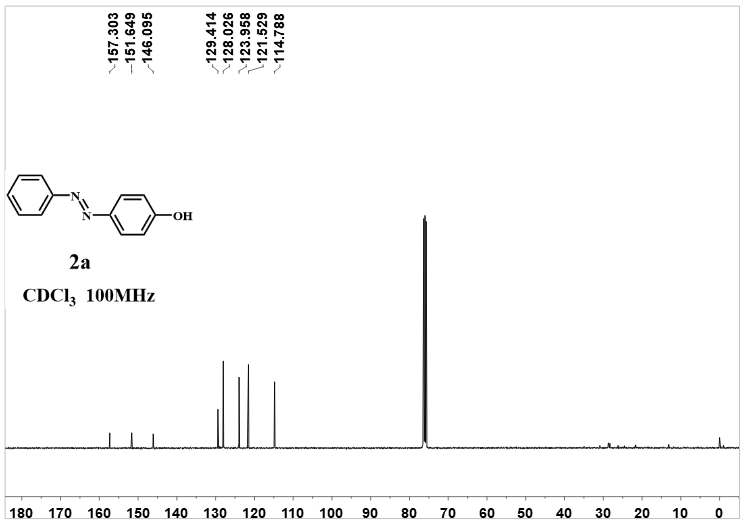


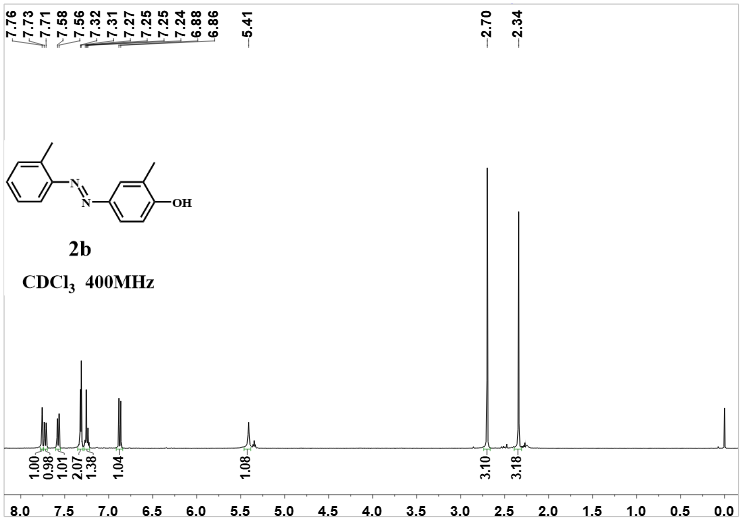


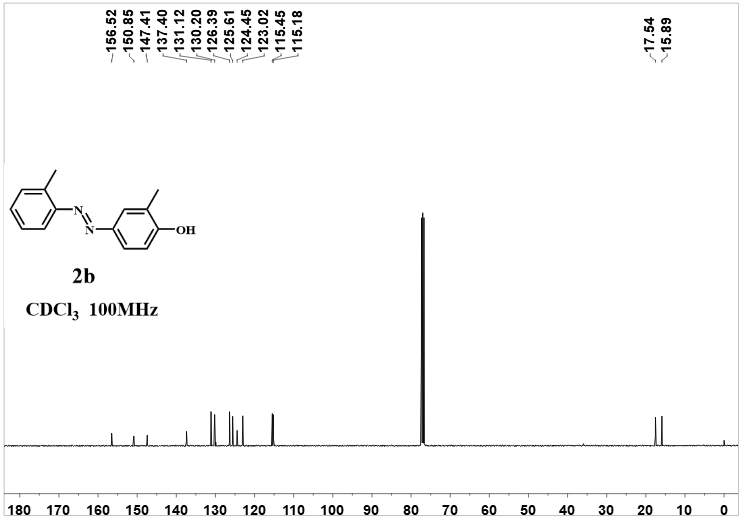


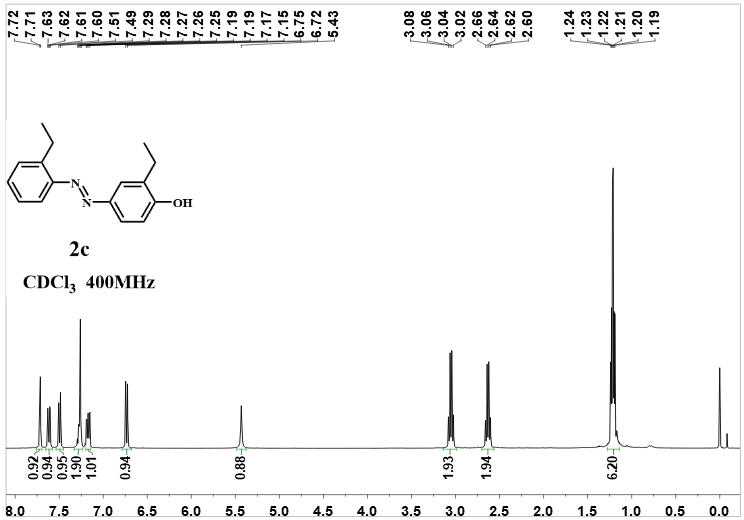


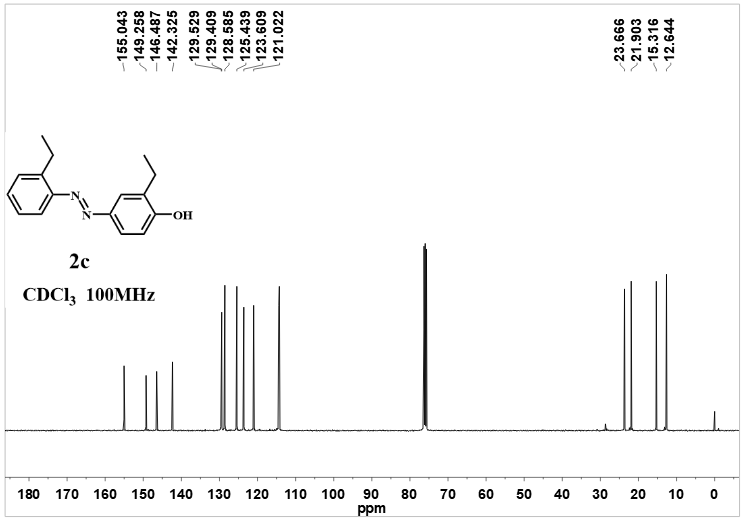


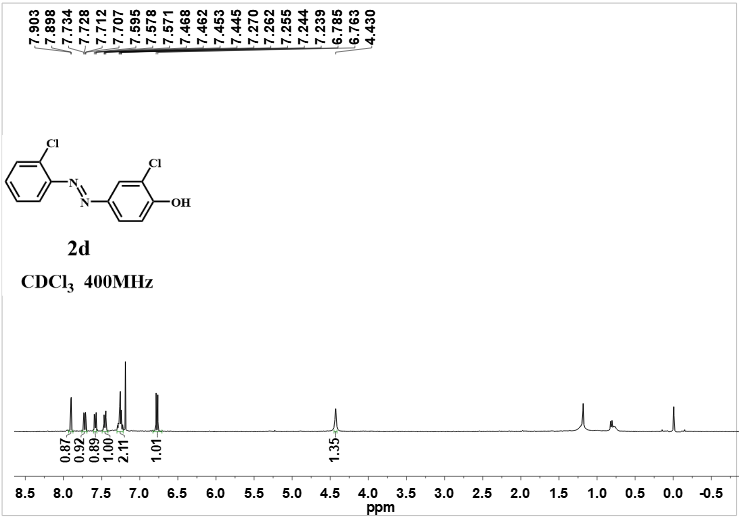


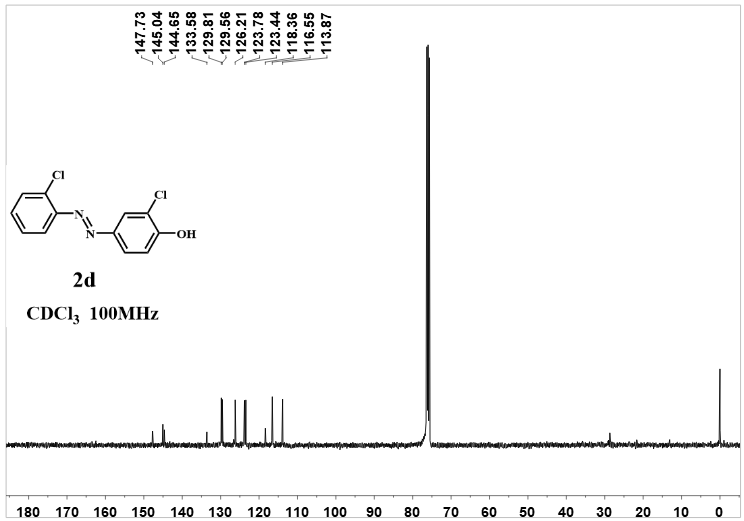


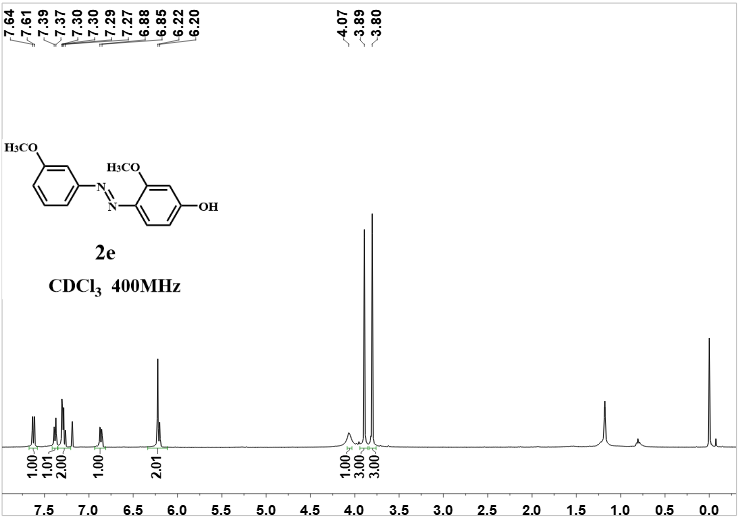


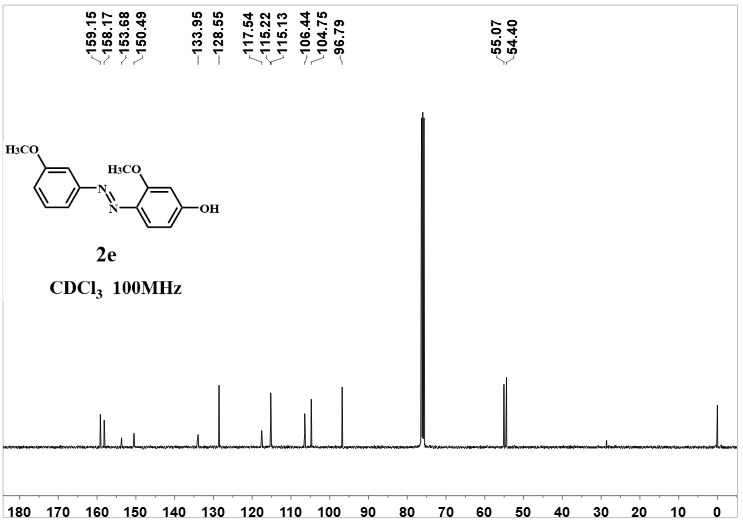


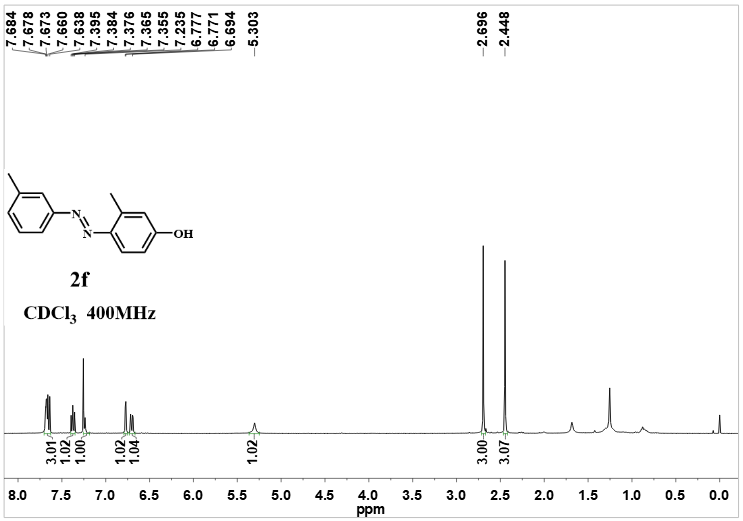


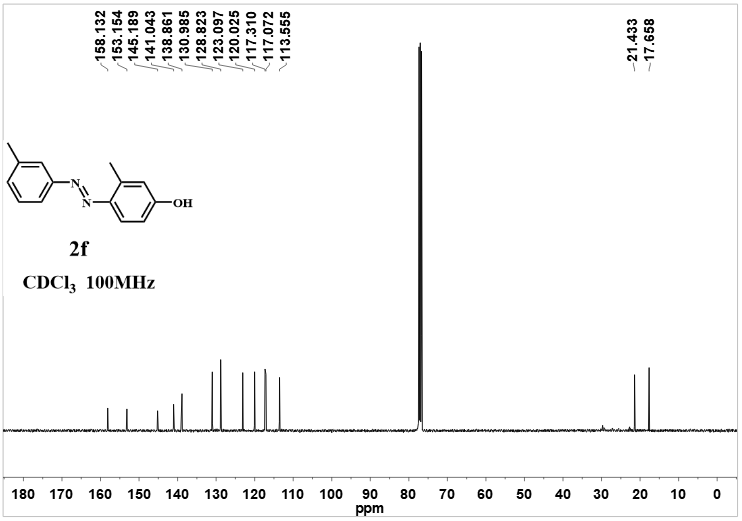


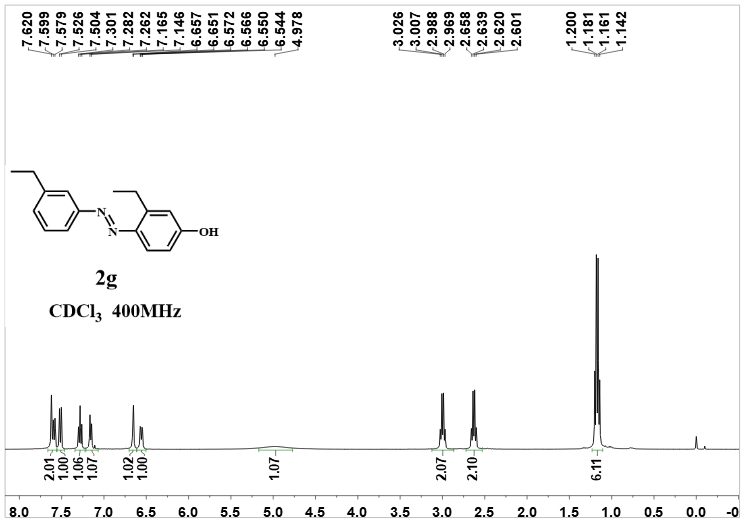


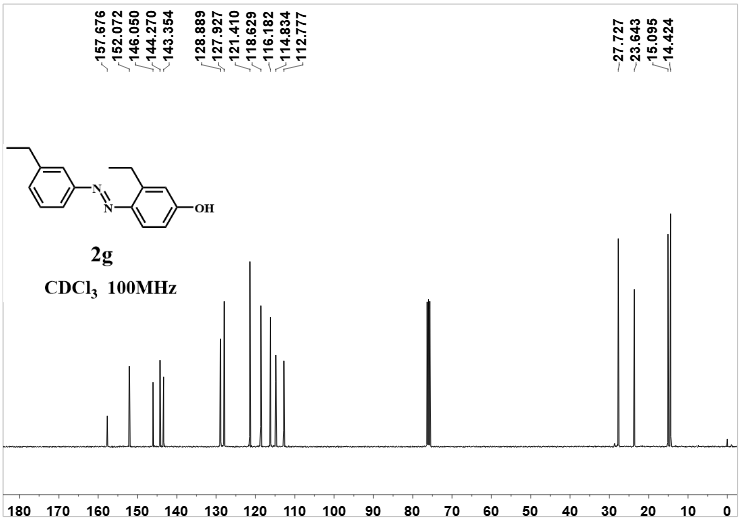


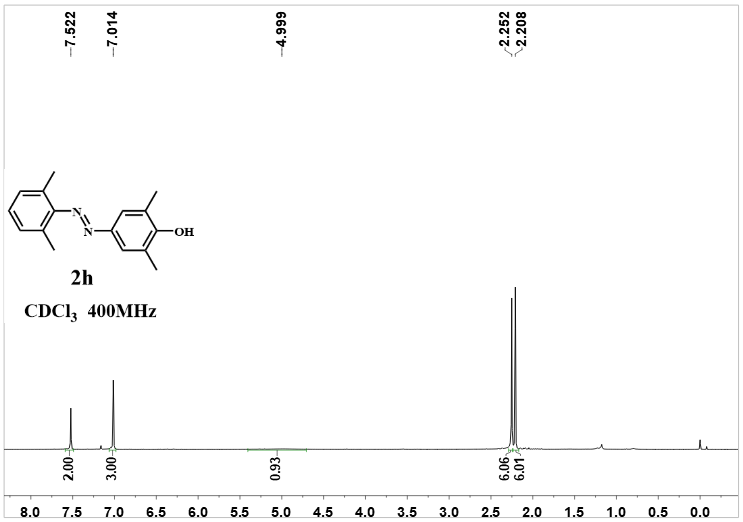


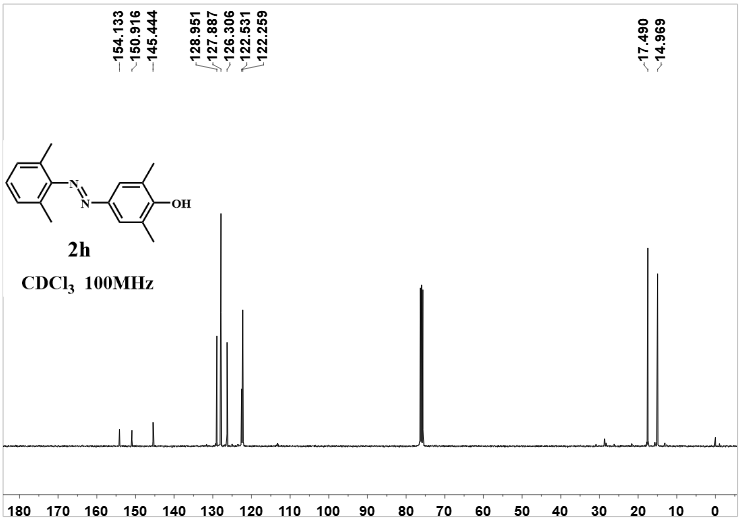


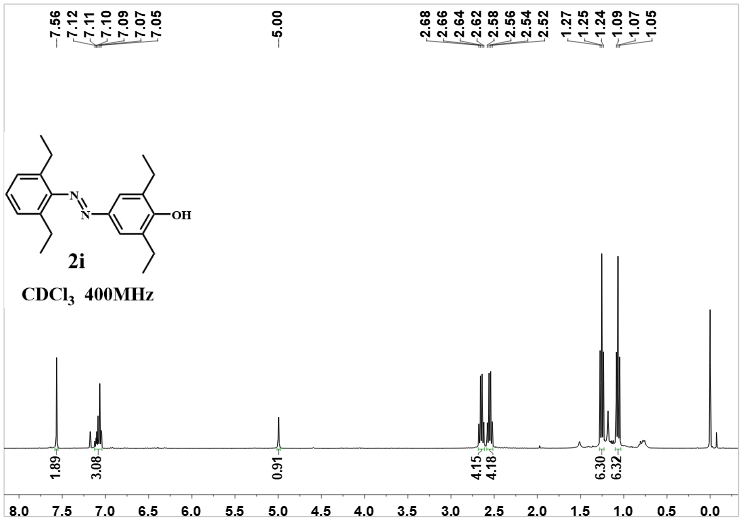


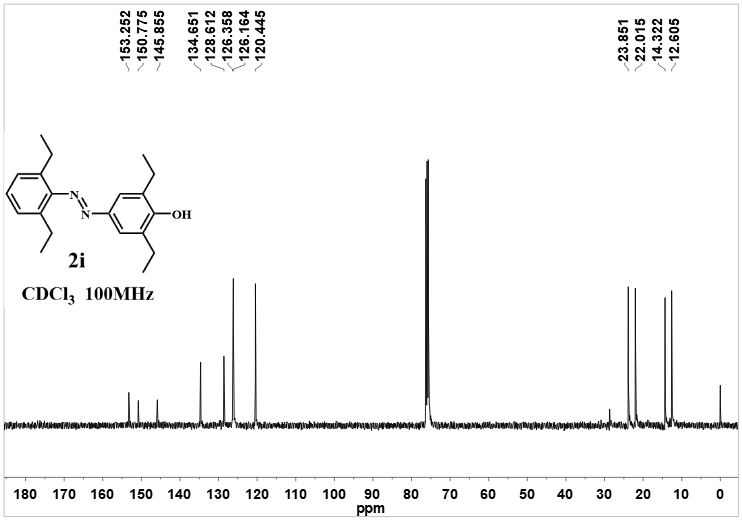


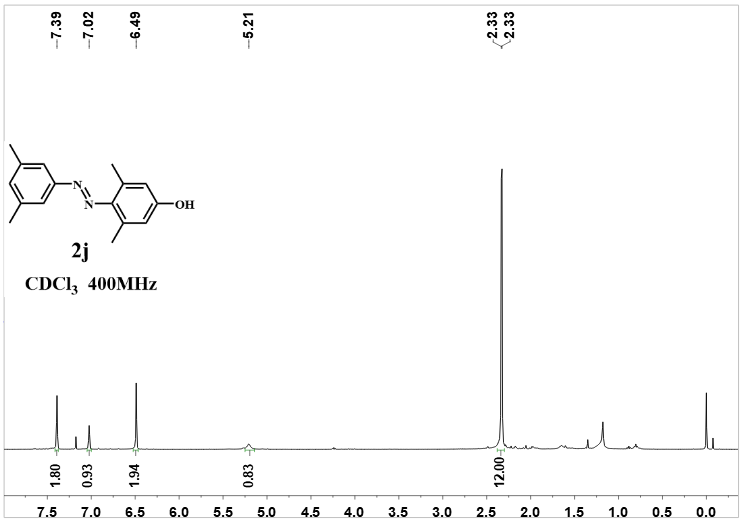


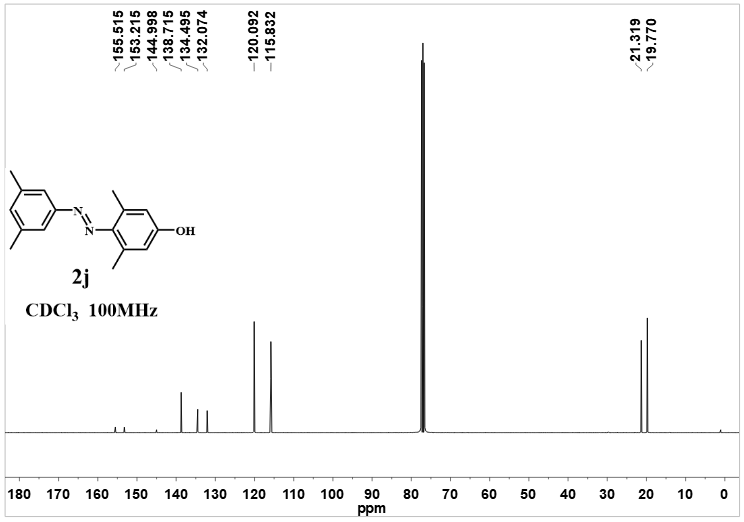


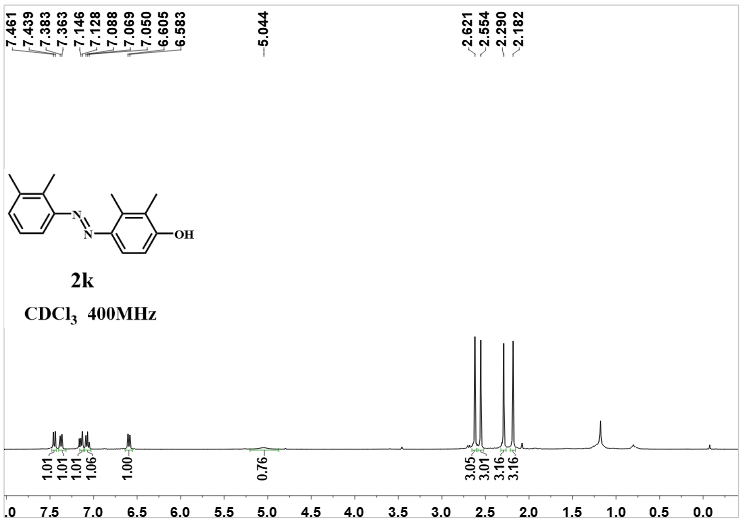


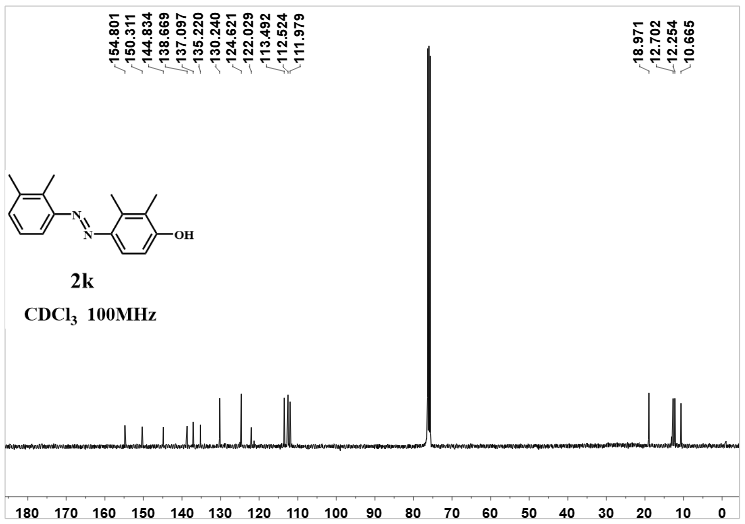


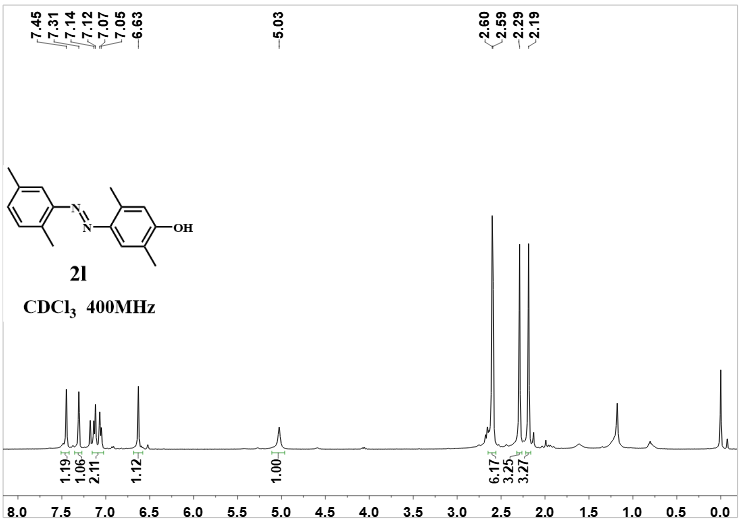


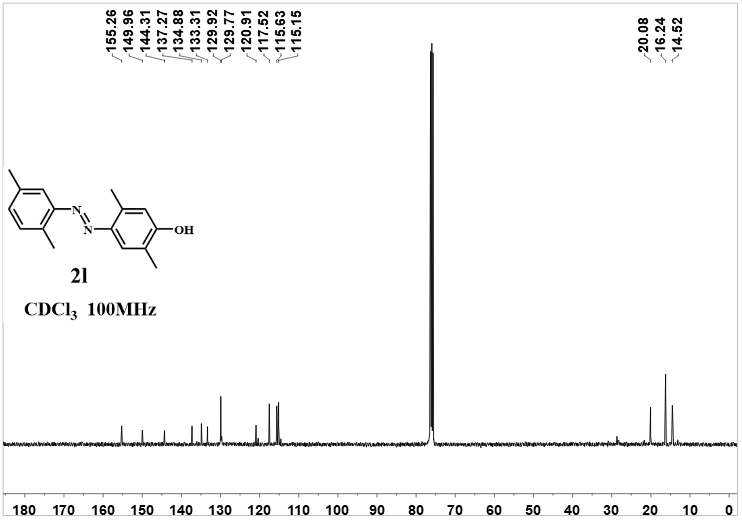


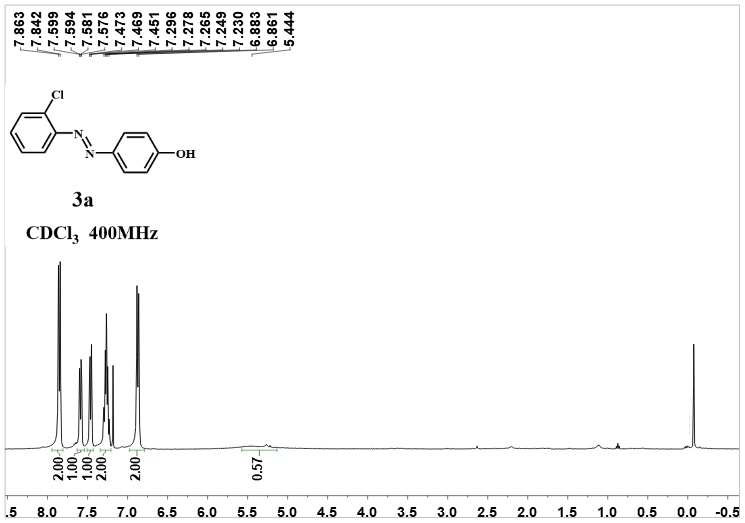


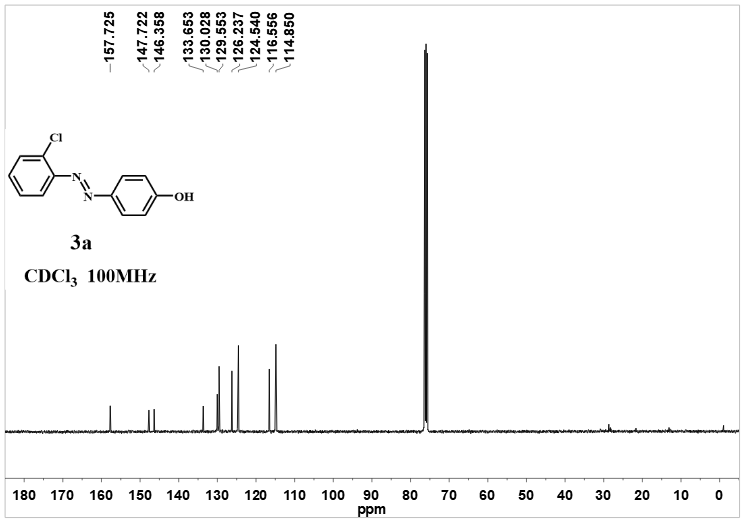


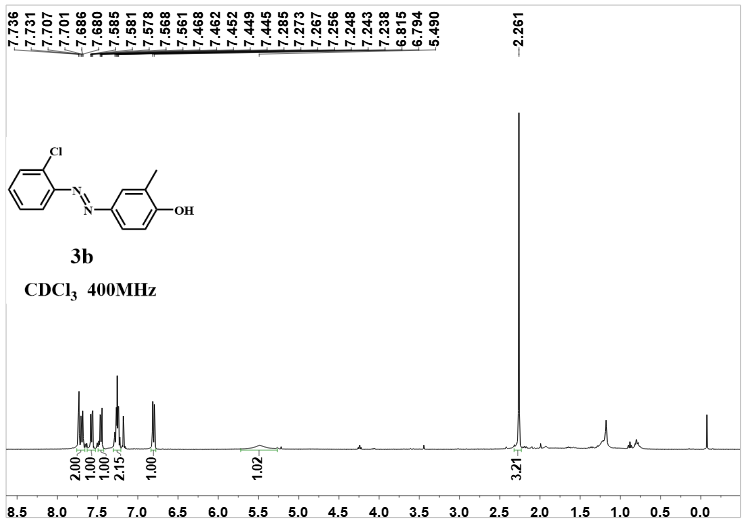


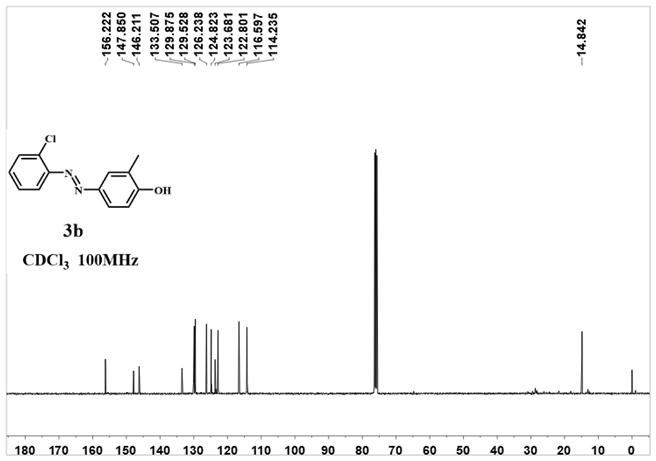


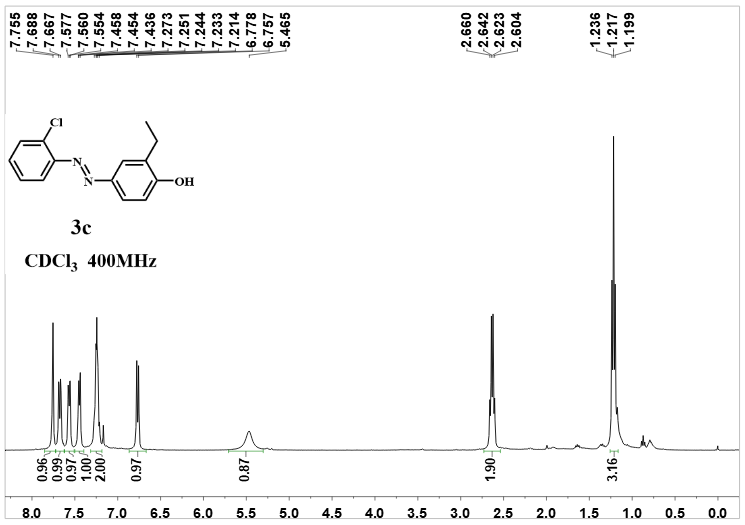


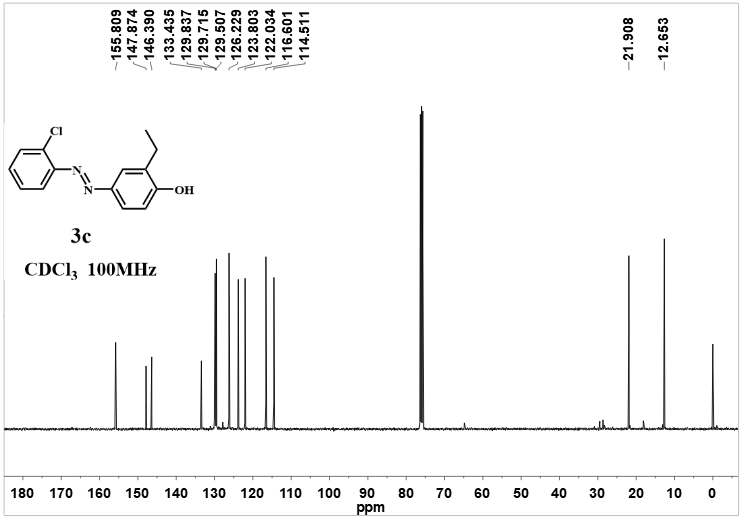


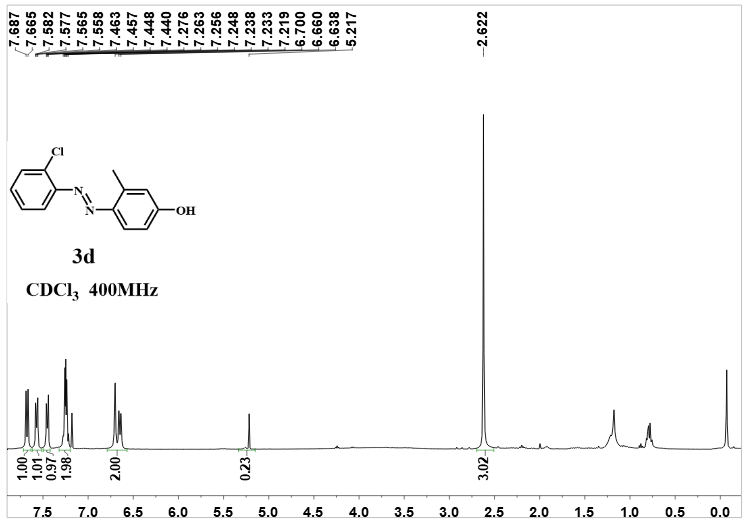

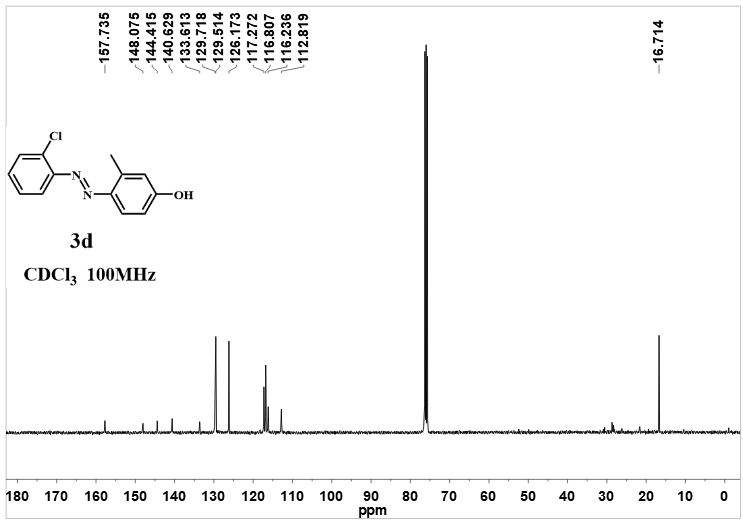


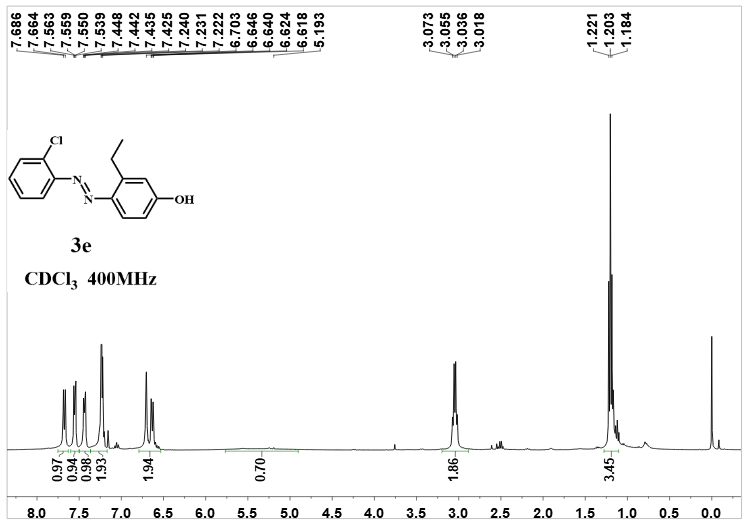


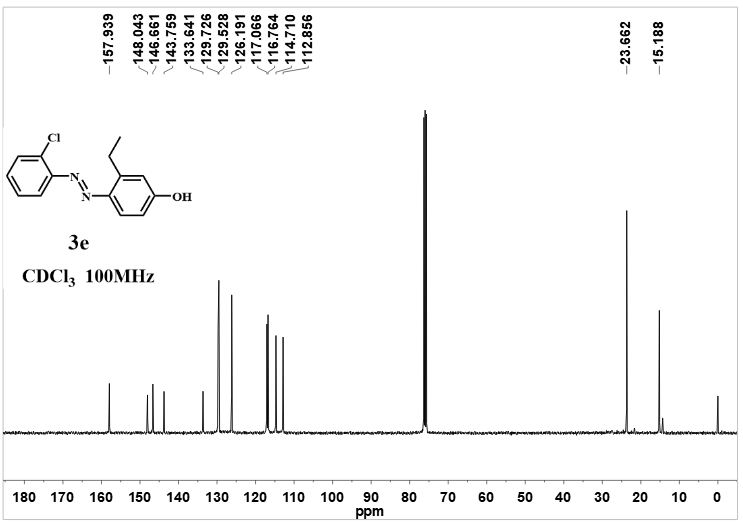


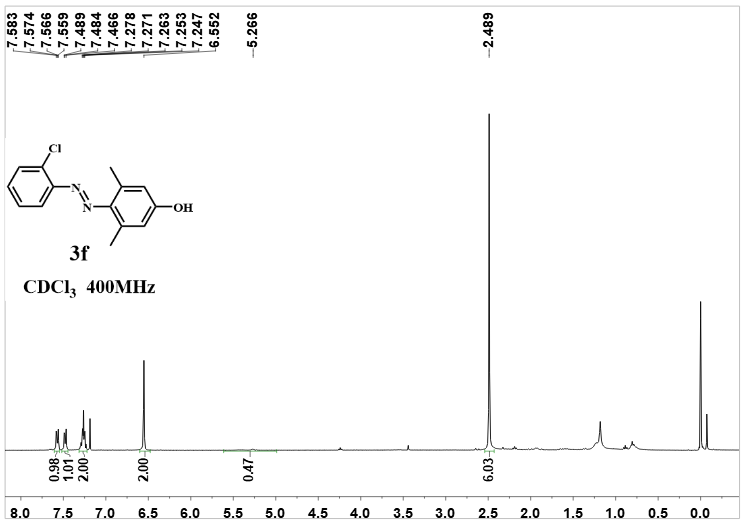


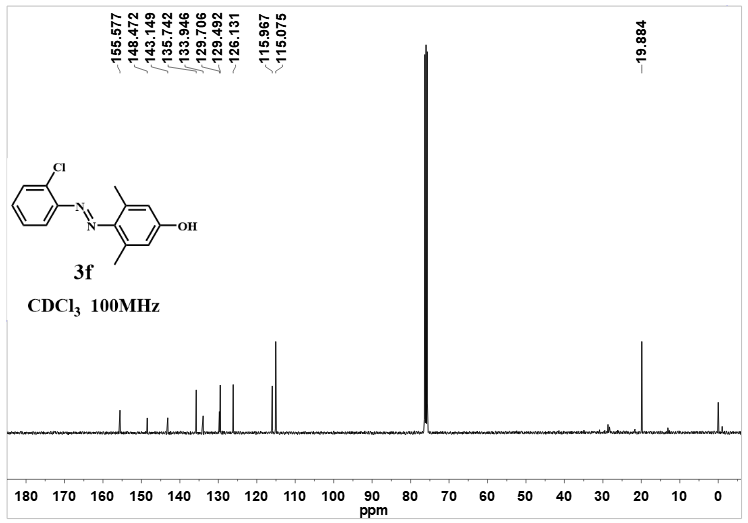


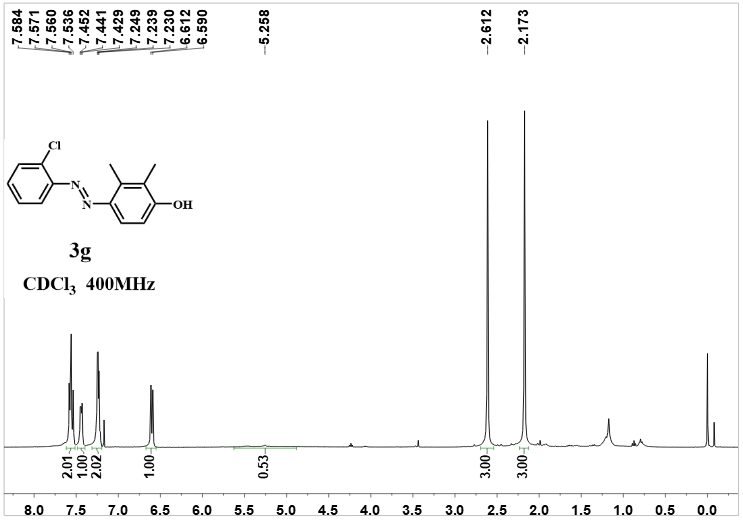


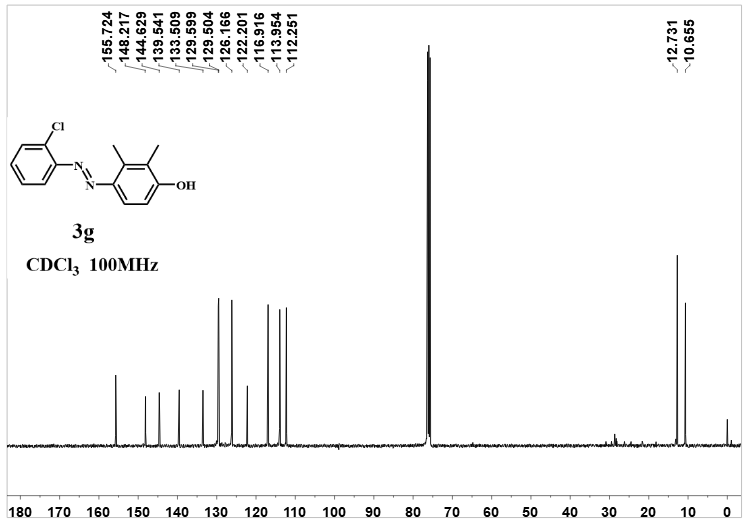


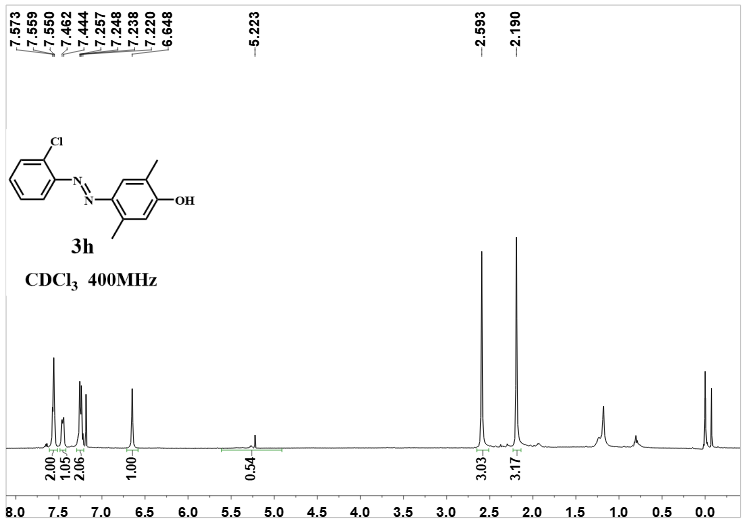


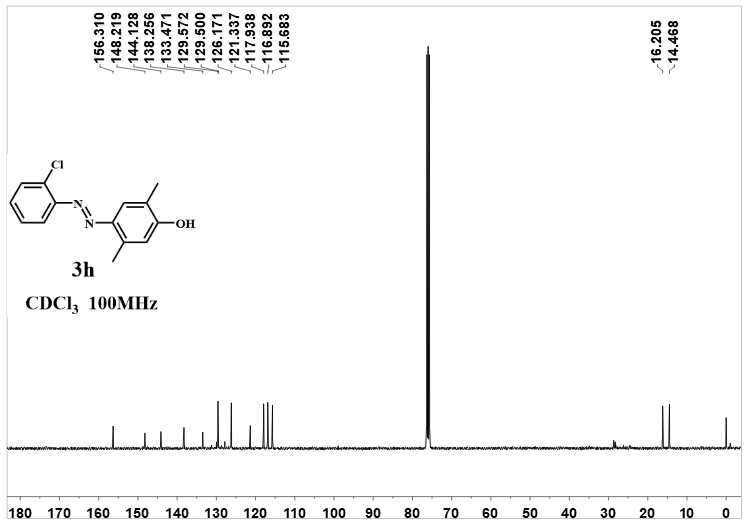


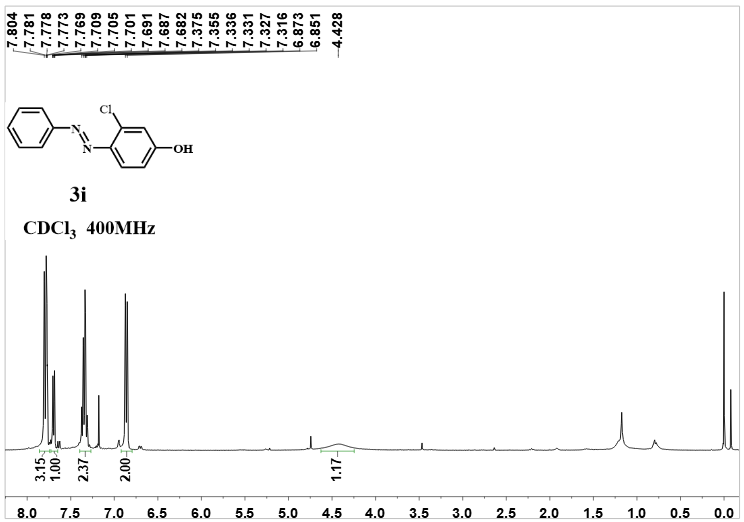


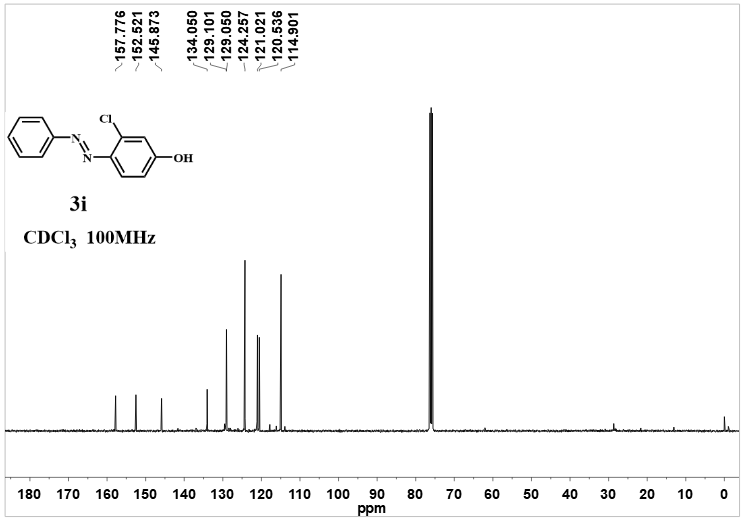


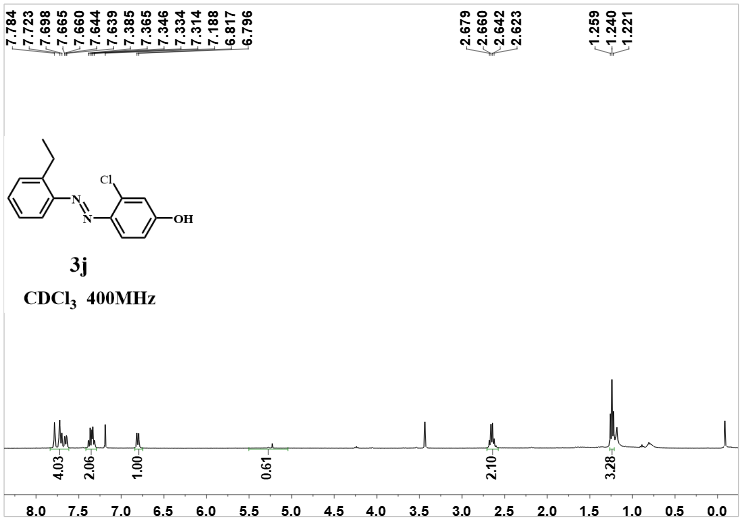


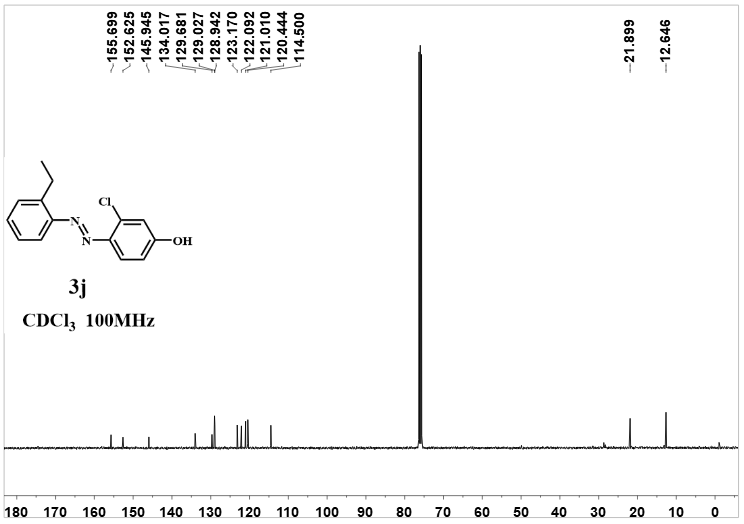


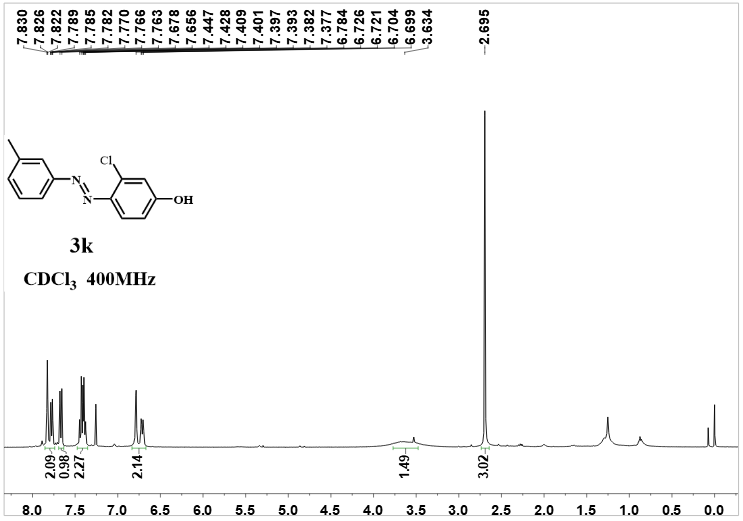


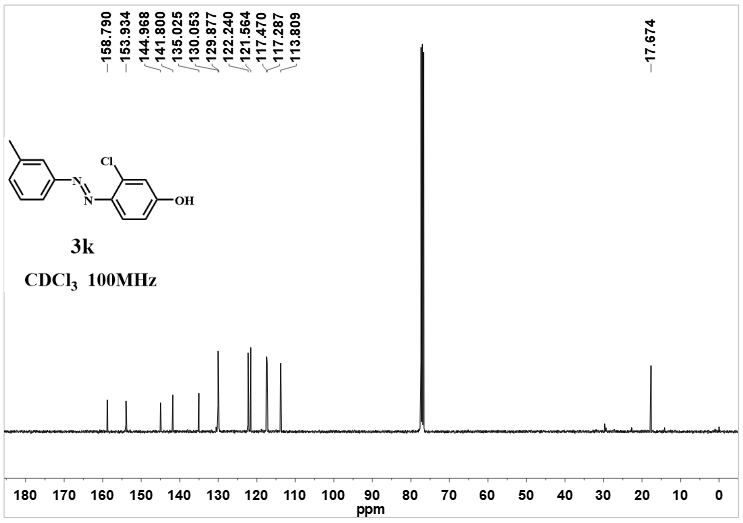


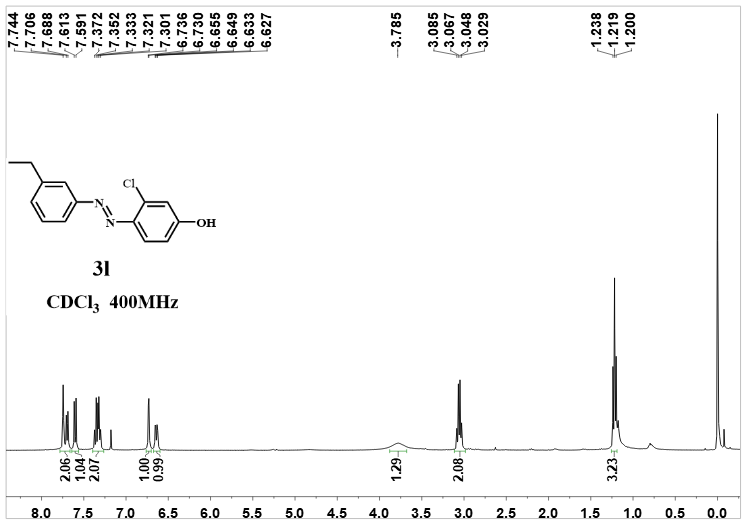


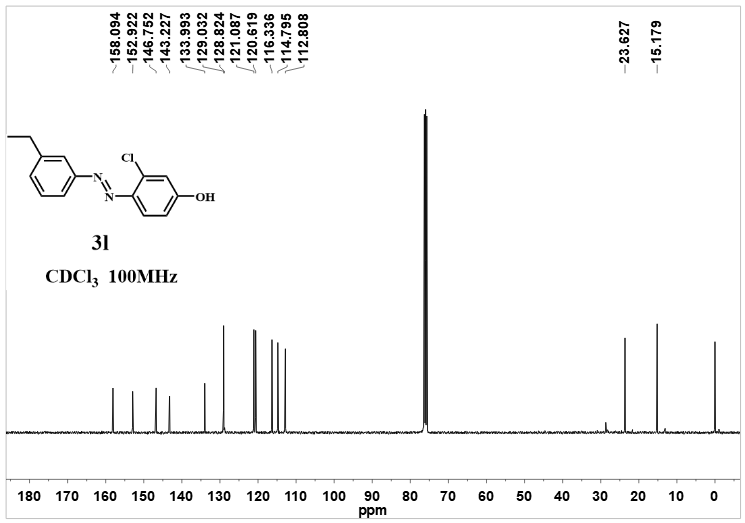


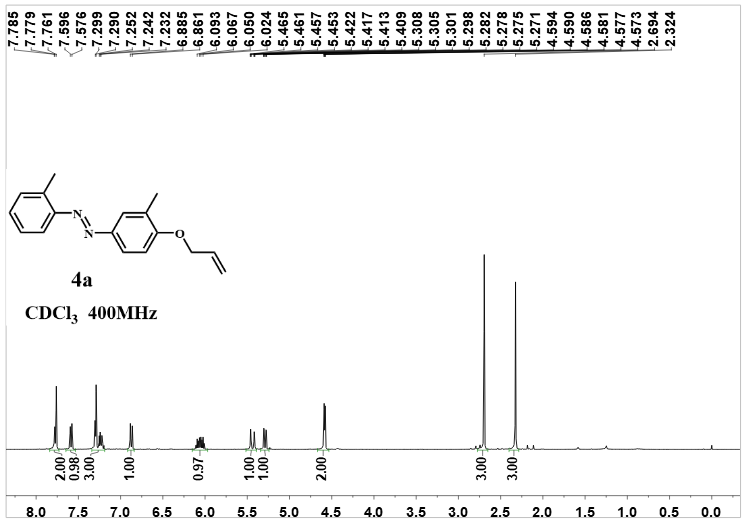


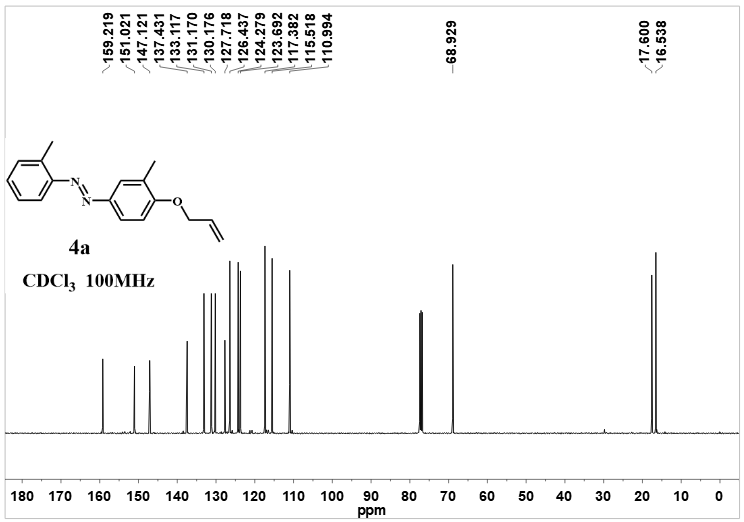


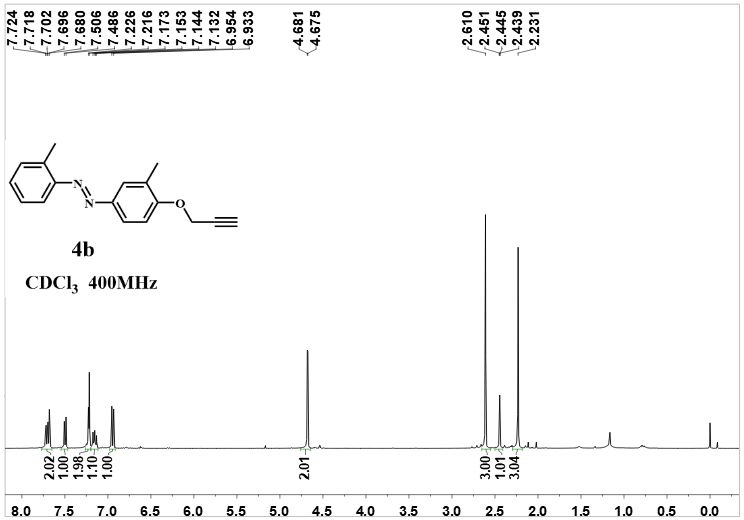


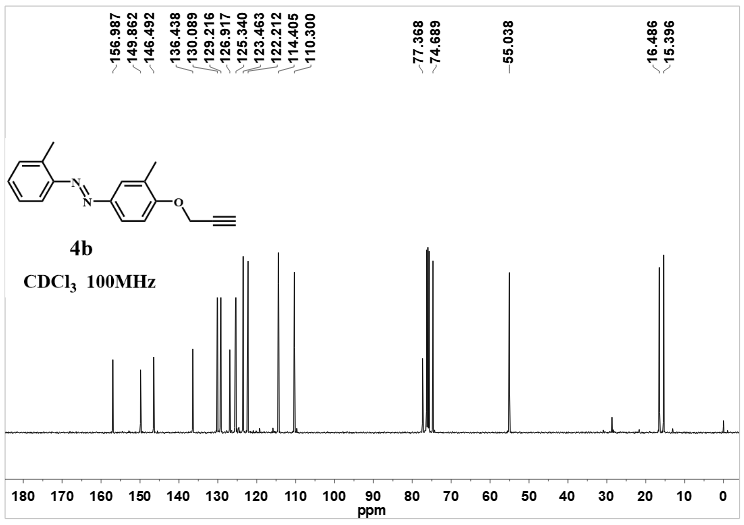


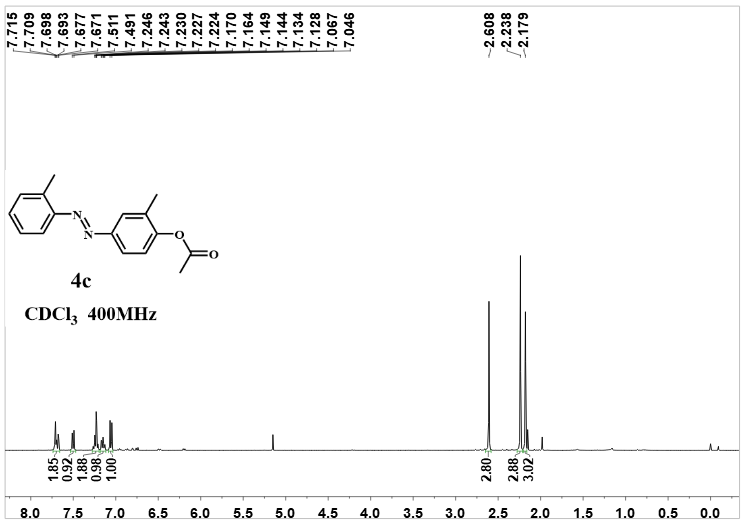


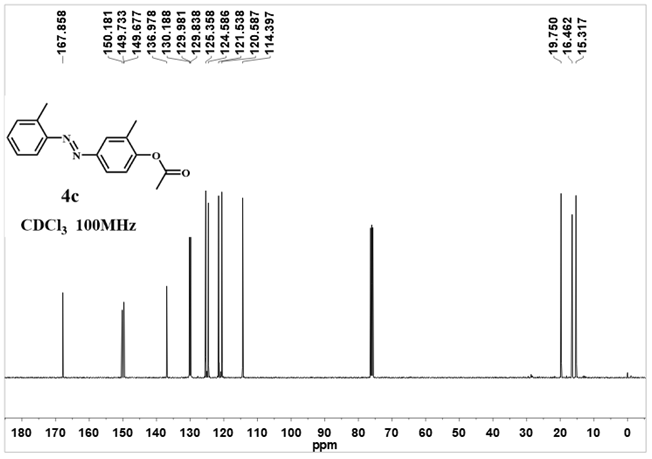

Supplement: Supplementary file 1 [file DataSheet1.docx]
